# A Survey on Medical Explainable AI (XAI): Recent Progress, Explainability Approach, Human Interaction and Scoring System

**DOI:** 10.3390/s22208068

**Published:** 2022-10-21

**Authors:** Ruey-Kai Sheu, Mayuresh Sunil Pardeshi

**Affiliations:** 1 Department of Computer Science, Tunghai University, No. 1727, Section 4, Taiwan Blvd, Xitun District, Taichung 407224, Taiwan; 2 AI Center, Tunghai University, No. 1727, Section 4, Taiwan Blvd, Xitun District, Taichung 407224, Taiwan

**Keywords:** eXplainable Artificial Intelligence (XAI), XAI recommendation system, XAI scoring system, medical XAI, survey, approach

## Abstract

The emerging field of eXplainable AI (XAI) in the medical domain is considered to be of utmost importance. Meanwhile, incorporating explanations in the medical domain with respect to legal and ethical AI is necessary to understand detailed decisions, results, and current status of the patient’s conditions. Successively, we will be presenting a detailed survey for the medical XAI with the model enhancements, evaluation methods, significant overview of case studies with open box architecture, medical open datasets, and future improvements. Potential differences in AI and XAI methods are provided with the recent XAI methods stated as (i) local and global methods for preprocessing, (ii) knowledge base and distillation algorithms, and (iii) interpretable machine learning. XAI characteristics details with future healthcare explainability is included prominently, whereas the pre-requisite provides insights for the brainstorming sessions before beginning a medical XAI project. Practical case study determines the recent XAI progress leading to the advance developments within the medical field. Ultimately, this survey proposes critical ideas surrounding a user-in-the-loop approach, with an emphasis on human–machine collaboration, to better produce explainable solutions. The surrounding details of the XAI feedback system for human rating-based evaluation provides intelligible insights into a constructive method to produce human enforced explanation feedback. For a long time, XAI limitations of the ratings, scores and grading are present. Therefore, a novel XAI recommendation system and XAI scoring system are designed and approached from this work. Additionally, this paper encourages the importance of implementing explainable solutions into the high impact medical field.

## 1. Introduction

XAI is recently dominating the research field for improving the transparency of the working model with the user. The brief history of AI development relates to statistical analysis, machine learning, natural language processing, computer vision, and data science. Even though such developments were present, it was not able to exceed human intelligence which was later progressed by neural networks, reinforcement learning, and deep learning. Such AI applications advancements were not only beneficial for weather forecasting analysis, self-driving cars, and the AlphaGo game capable of competing with the best humans’ skills, but also were found to be of critical importance within the medical domain and its progress [1,2]. Human–Computer Interaction (HCI) research is also progressing to automate many applications and provide solutions [3]. Nevertheless, the improvements within the life expectancy have been recently improved with the use of advanced technologies and still will be beneficial to tackle the problems faced within different categories of the medical domains. Therefore, developments within the medical domain are discussed which focuses mainly on pneumonia status, bloodstream infections (BSI), acute kidney injury (AKI) and hospital mortality (HM) prediction [4]. XAI is necessary to be evaluated with the medical domain progression as it provides complete details of each algorithmic step thought to be trusted within the medical domain, practitioners, and experts. The three stages in XAI can be given as (i) explainable building process for facilitating acceptance, (ii) explainable decisions for enabling trust with users and administrators, and (iii) explainable decision process for the interoperability with business logic [5]. The goal of XAI is to provide machine and deep learning algorithms for better performance with explainability, which further allows ease of user trust, understanding, acceptance, and management.

Even though the drawbacks of the previous AI system including black box models, catastrophic consequences in medical diagnosis were discussed by some reference [6] but later by the progression with the model development, enhancement and tuning high accuracy, quality of work, and speed was achieved. XAI was also found to be the European Union’s General Data Protection Regulation (GDPR) standard complaint, as no data is revealed to the outside system/participants by disclosing private medical datasets and providing explanations in the decision process.

### 1.1. Motivation

The motivation for this work is thought from realizing “Why explainability is necessary in the medical domain?”, or it can also be given as the actual motivation is the laws and ethics aspects in the applications of XAI need to be considered before they can be applicable in the medical domain. In various parts of the world, the right to explanation is already enshrined by law, for example by the well-known GDPR, which has huge implications for medicine and makes the field of XAI necessary in the first place [7]. The medical AI is termed as a high-risk AI application in the proposal by European legislation, which is regulated for the fundamental rights of human dignity and privacy protection. In this case, the decision is based solely on real-time AI processing after the decision to assess, which is overcome by the “right to explanation”. As the GDPR prohibits decisions solely based on automated processing, the final decision is drawn from the human in the loop approach and informed consent of the data subject. The legal responsibility of medical AI malfunctioning leads to civil liability instead of criminality. Additionally, compulsory insurance is required in the future against the risks of AI applications by the liability law. The ethics in medical AI gives a sustainable development goal for the “good health and well-being” by the United Nations [8]. The bias or flaw in training data due to the societal inclination impact may lead to the limitations in AI performance. Therefore, the factors given by the ethics committee discussions about the contribution of medical AI needs to be given so as to know the specific part decision/action, communication by AI agent, the responsibility taken by the competent person, transparency/explainability, method reference, avoiding manipulation for high accuracy, avoiding discrimination, and the algorithm must not control AI decision and actions. The purpose is to make AI a friend, and combining all of the above responsibilities, it would be termed as XAI. Therefore, the XAI approach provided within this paper constitutes one of the major portions for the directions of future approach and perspective.

### 1.2. Interpretability

A recent survey on medical XAI focuses completely on interpretability [9]. As the medical field possessess a high level of accountability and transparency, a greater interpretability is needed to be explained by the algorithm. Even though the interpretability is treated equally across all the hospitals, it should be handled with caution; medical practices should be the prime focus for interpretability development, and data based on mathematical knowledge for technical applications are encouraged. The different interpretability categories referenced here are perceptive and mathematical structures. The perceptive interpretability is mostly a visual evidence that can be analyzed using saliency maps, i.e., LIME, Class Activation Map (CAM), Layer-wise Relevance Propagation (LRP), etc. In signal methods, the stimulation/collection of neurons are detected, i.e., feature maps, activation maximization, etc. The verbal interpretability is the human understandable logical statements based on the predicates, connectives, i.e., disjunctive normal form (DNF) and NLP. The mathematical structure based interpretability is the popular mechanism used through machine learning and neural network algorithms, whereas the predefined models are the relation between variable to output variable that includes logistic regression, Generative Discriminative Machine (GDM), reinforcement learning, etc. Ultimately, the feature extraction from the input source is performed by graphs presentation, clustering, frame singular value decomposition (F-SVD), etc.

### 1.3. Feedback Loop

A feedback loop designed for the XAI continuous development includes multiple phases, which can be given as follows [10]. The model debugging and visualization is performed first, then model compilation is performed by testing, after which the model is then released based on versioning. During the output phase, the predictions are performed by explainable decisions in which different models are compared for analysis and performance monitoring is performed successively followed by debugging and feedback loop. The model’s explainability increases based on how much it supports open box architecture. The deep learning models, i.e., convolutional neural networks (CNN), recurrent neural networks (RNN) are the least explainable and are the predecessor of ensemble model, i.e., random forest, XGB. The statistical models and graphical models are easy to understand and are more straight forward, i.e., SVM, Bayesian brief net, Markov models, etc. The decision trees, linear models, and rule-based models are the most explainable and completely open box architecture models. The different XAI categories explained within this reference include dimension reduction which are presented as most important input features by selecting optimal dimensions, e.g., optimal feature selection, cluster analysis, LASSO, sparse deep learning, and sparse balanced SVM. The feature importance is used to capture characteristics and correlation amongst features for XAI models, e.g., feature weighting, DeepLIFT, SHAP, whereas the attestation mechanism captures the important areas where attention is required by the model, e.g., MLCAM, CAM, GRAD-CAM, Respond-CAM. The XAI well-known knowledge distillation is drawing the knowledge from a complicated model to a more rationalized model, e.g., rule-based system, mimic learning, fuzzy rules, and decision rules. Ultimately, the surrogate models are the locally faithful models and approximate reference models to surrogate model, e.g., LIME, LRP, etc.

### 1.4. General XAI Process

As the XAI necessity is thought to be effective for improvements within the future system. Therefore, the initial steps required for the XAI process are as follows:

(a) Pre-processing: The data cleaning, recovery/imputation and top feature analysis are described in this phase. The data cleaning refers to the handling of the incorrect, duplicate, corrupted, or incomplete dataset, whereas the data imputation refers to the substitute values for replacing missing data. In case of SHapley Additive exPlanations (SHAP), which is a part of game theoretic approach for identifying the top dominating features to help achieve better prediction results [8].

(b) Methodology: The model specifically designed for the effective implementation of the machine or deep learning construction and tuning. There are many machine learning algorithms, i.e., naïve bayes, linear regression, decision trees, support vector machine (SVM), etc., whereas neural networks are used to mimic human brains by providing a series of algorithms for recognizing relationships within the dataset [9,10]. The interpretable deep learning refers to the similar concepts except inspecting data processing at each layer and thus helping the designer to control the data movement and mathematical operations within it. Furthermore, the layers can also be configured by setting the feature learning by convolution, max pooling, and classification by fully connected, activation functions, etc.

(c) Explanation: This phase provides the explanation for each decision transparently to know the importance and action taken by the algorithm. The explanation provides detailed reasoning for all the decisions taken within the model from preprocessing, algorithm for prediction, classification, evaluation, and conclusion. As the explanations form the crucial content of XAI, it improves the acceptance of the deployed system to the end user, domain experts, or clients.

(d) Re-evaluation: Feedback system designed to understand limitations as the difference in choices made by the users and the algorithms. At the end of the algorithm, the end user can interact with the system by providing the necessary feedback for each decision and parameters used, which later can be evaluated effectively by re-configuration in the successive version. Therefore, it not only promotes ease of usage but also makes the end user as the part of the system, which can improve the next version of the training data and weights enhancement.

### 1.5. Objectives

The objectives for this survey can be given as follows:

- Determine the current progress within the different infection/diseases based on AI algorithms and their respective configurations.
- Describe the characteristics, explainability, and XAI methods for tackling design issues in the medical domain.
- Discuss the future of medical XAI, supported by explanation measures by human-in-the-loop process in the XAI based systems with case studies.
- Demonstrate a novel XAI Recommendation System and XAI Scoring System applicable to multiple fields.

A paper plan for this survey is given as follows: related works describe the various infections/diseases-based references, methods, and evaluations in Section 2; the difference between AI and XAI methods is given in Section 3; and recent XAI methods usage with its importance in Section 4. Afterwards, the characteristics of XAI0-based explanation in Section 5; future of XAI explainability in Section 6; and prerequisite for AI and XAI explainability in Section 7. Lastly, details about the case study for application usage in Section 8; XAI limitations in Section 9; XAI Recommendation System in Section 10; and XAI Scoring System in Section 11, followed by the conclusion and references.

## 2. Related Works

In this section, we are going to present the background for the medical domain with respect to the various infection or diseases related works, which are recently presented as a solution using AI or XAI. The research work presented in medical fields is mostly evaluated using mathematical statistics and machine learning algorithms as given in Table 1, Table 2, Table 3 and Table 4. Therefore, it presents several opportunities to provide XAI-based implementation and improve the current understanding with better evaluation using classification.

The highly affecting acute respiratory disease syndrome (ARDS) or pneumonia-based evaluation supports various features such as vital signs and chest X-rays (CXR) [11]. The classification in this case can be mostly performed within the combination or independent data sources of vital signs and/or CXR. Usually the patients within this case are required to be first identified with specific symptoms of cough, fever, etc., and then the vital signs and/or CXR are used by the medical examiners to diagnose and know the healing progress of the pneumonia status. Later, the discharge is predicted using this work, and also more detailed configuration can help to understand the algorithm behavior. The mechanism for local determines a single decision system, whereas for global it determines multiple decisions.

**Figure 1:**
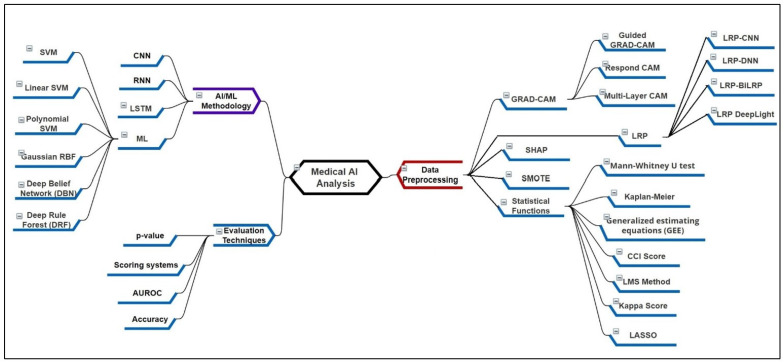
MindMap diagram for the medical AI analysis.

Figure 1 presents the mindmap diagram for the literature survey analysis. The explanation type ante-hoc is for open/human understandable models and post hoc for black boxes and Deep Neural Networks (DNN). One of the commonly occurring infections within patients is bloodstream infection (BSI), which can be identified by the presence of bacterial or fungal microorganisms within the blood samples [21]. It is also popularly known as sepsis/septic shock and has severe symptoms. The most common symptoms include fever, increase in heart rate, high blood pressure, chills, and gastrointestinal issues. In the previous studies, the BSI was studied in detail with vital signs and laboratory variables with ICU admission data. The preprocessing is mostly done to recover the missing data in the BSI and Non-BSI cases, which is later evaluated using the machine learning model. The BSI once detected then later can be cured using medicine treatment.

A severe type of infection or condition, which can be caused by multiple factors affecting blood flow to the kidney or medications side effects is known as acute kidney injury [31]. The symptoms can be basically seen in the lab tests, which include urine output and serum creatinine levels. In case of ventilation support, additional parameters are considered for the features. The preprocessing could help to improve data quality and provide promising results. Machine learning has shown to identify the stage and level of AKI, which has helped to apply proper medication treatment, recovery for the mild and control the severe conditions. In case of comorbidities or critical conditions, the hospital mortality is thought to be an important prediction [41]. There are more features available for such cases, as it involves distinct ICU parameters. Additionally, the medication courses and its related effects are available. The criteria for considering critical cases is the first filter for preprocessing and later data imputation can be added, if necessary. Previously, many works have provided such predictions using time windows before 48, 72 h, etc. by using either statistical, machine learning, and/or CNN methods. Such work is important in case of shifts in the medical treatment department or medication course.

## 3. Potential Difference between the AI and XAI Methods

**Figure 2:**
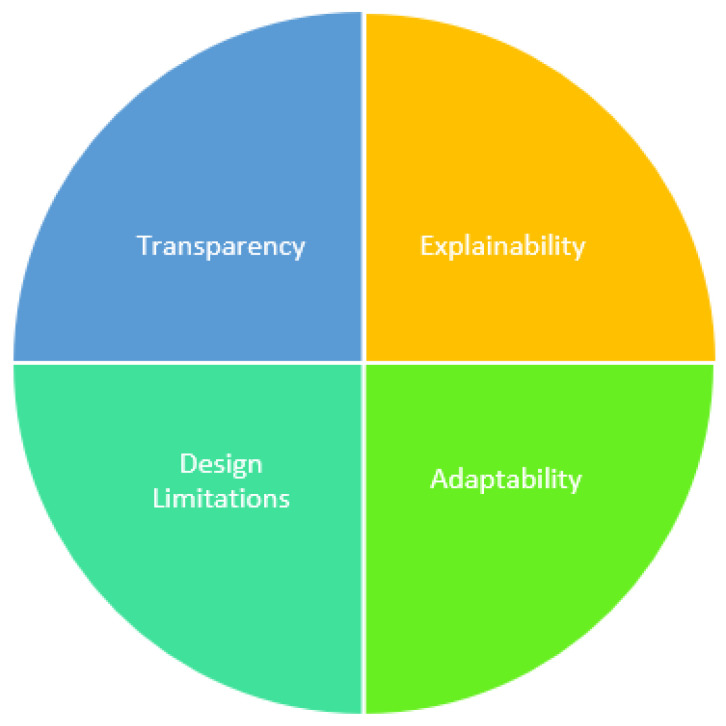
Difference in AI and XAI Methods.

Figure 2 presents various factors responsible for the difference in AI and XAI methods.

Researchers are required to select XAI methods for the benefits as discussed below:

### 3.1. Transparency in the System Process

The use of conventional black-box-based AI models have limited its use and transparency of the system. Therefore, XAI methods are known for their transparent systems that provide the details of the data preprocessing, model design, detail implementation, evaluation, and conclusion. Transparency provides the user with complete system design that can be later configured, improved, versioned, and audited effectively.

### 3.2. Explainability of the System

The AI model lacks explainability for the system process. Therefore, the user’s trust can be gained by a highly explained XAI-based decision process. The decision taken on every step of the system process and its supporting explanation makes it more effective. In case of model design issues, the explainability can also help to identify at which process step the erroneous decision was made and thus later can be resolved. The explainability is crucial for the initial data analysis, decision, and action for the whole XAI model.

### 3.3. Limitations on the Model Design

The AI models are usually black box and are not accessible to the end users. In comparison, the XAI provides models more interpretability at each structural layer, which is used to know the data quality, feature distribution, categorization, severity analysis, comparison, and classification. Thus, the acceptance of the XAI models is more due to interpretability. The user is more confident and has trust in the system. Nevertheless, false positive values can also be caught and analyzed in detail to avoid system failure and better treatment.

### 3.4. Adaptability to the Emerging Situations

The XAI models are known for high adaptability by using the feedback technique. The domain experts/medical examiners may be interested in applying/modifying a new feature. In severe cases, ICU parameters can also be adopted for better discharge and mortality classification. Due to recent infections, the cases of comorbidities are on the rise and such complex cases need high adaptability and explainability for the treatment. Ultimately, the model quality can be kept consistent and will be applicable to long-term usage.

## 4. Recent XAI Methods and Its Applicability

**Figure 3:**
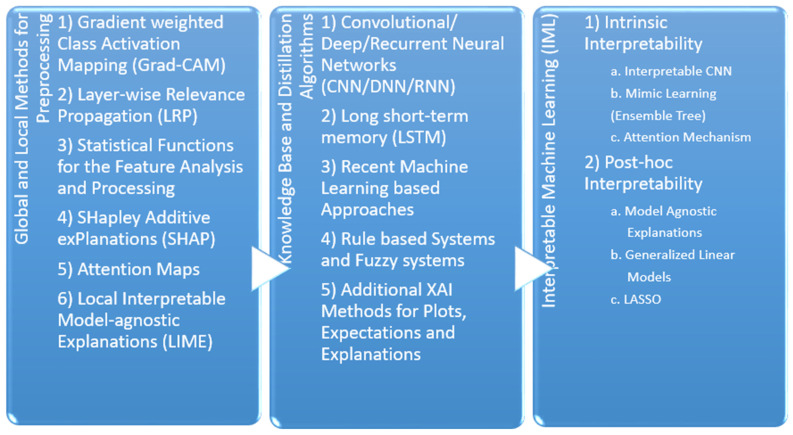
XAI Methods Categorization.

The recent reference papers show the approach of providing interpretability and transparency of using the models as shown in Figure 3. Even though the models, dataset, criteria, and outcome are specified in detail in many medical domain papers, still the explainability and justifiability needs to be provided for every case. In the future, interactive AI systems will be in more demand for providing such explainability and interaction with the domain experts to continuously improve the outcome, which is adapted to various situations such as changes in human, weather, and medical conditions. The tables from 1 to 4 are probable approaches for the respective infection/disease and are deemed to be appropriate for the hospital-based recovery prediction. For this section, we are going to discuss the preprocessing methods used for the recent paper, algorithms used within their respective models, and outcome.

### 4.1. Local and Global Methods for the Preprocessing

#### 4.1.1. Gradient Weighted Class Activation Mapping (Grad-CAM)

Grad-CAM [11] is used for prediction of the respective concept by referring to the gradients of the target, which is passed to the final convolutional layer. The important regions are highlighted using the coarse localization mapping. It is also known to be a variant of heat map, which can be used by image registration to identify the different image sizes and scales for the prediction. Grad-CAM is a propagation method, easy to visualize and provides user-friendly explanations. It is one of the popular methods in object detection and is recently used frequently within the medical domain to identify different diseases and affected areas of the patient. The chest X-ray (CXR), CT-scan, brain tumors, fractures in the different human/animal parts can be easily highlighted by such application. As the accuracy with sensitive domain is not recommended, there are several other versions for the CAM supported analysis include Guided Grad-CAM [12], Respond-CAM [13], Multi-layer CAM [14], etc. The Guided Grad-CAM is used to check models prediction by identifying salient visual features. Thus, the interest class relevant features are highlighted by the saliency maps. The Grad-CAM and guided backpropagation pointwise multiplication is known as saliency maps. The Guided Grad-CAM is known to generate class specific maps, which are the last convolutional layers feature map dot product and neurons combining to a predicted class score by partial derivatives. The Respond CAM is used to operate on the 3D images having complex structures of macromolecular size from the cellular electron cryo-tomography (CECT). The Respond-CAM has a sum to score property for better results than Grad-CAM and is used to highlight 3D images’ class discriminative parts using weighted feature maps.

The Respond-CAM’s sum-to-score property can be given as $y^{\left(c\right)}$ as the class score, $b^{\left(c\right)}$ is the last layer CNN parameter, $\underset{i,j,k}{\sum }\left(L_{A}^{\left(c\right)}\right)$ is the class c sum for Grad-CAM/Respond-CAM and C as the number of classes given in Equation (1).

(1) $$y^{\left(c\right)}=b^{\left(c\right)}+\sum_{i,j,k}{\left(L_{A}^{\left(c\right)}\right)}_{i,j,k}$$

The Multi-layer Grad-CAM is used to compute conditional probability of the selected feature with a single maxout hidden layer. It is based on maxout units, a single hidden layer with a softmax function to normalize output probability.

#### 4.1.2. Layer-Wise Relevance Propagation (LRP)

It is also one of the popularly used propagation methods, which operates by using the propagation rules for propagating the prediction backward in the neural network. The LRP can flexibly operate on input such as images, videos, and texts. The relevance scores can be recorded in each layer by applying different rules. The LRP is based and justified using a deep taylor decomposition (DTD). It can be set on a single or set of layers in the neural network and can be scaled in the complex DNN by providing high explanation quality. It is also popularly used in the medical domain consisting of CXR, axial brain slices, brain relevance maps, and abnormalities, etc. The versions available in LRP are LRP CNN, LRP DNN, LRP BiLRP, LRP DeepLight for the heatmap visualizations. The LRP relevance is higher as compared to other visualization/sensitivity analysis. The input representations are forward-propagated using CNN until the output is reached and back-propagated by the LRP until the input is reached. Thus, the relevance scores for the categories are yielded in LRP CNN [18]. For the LRP DNN [19], the CNN is tuned with initial weights for the activity recognition with pixel intensity. In LRP BiLRP [20], the input features pairs having similarity scores are systematically decomposed by this method. The high nonlinear functions are scaled and explained by using composition of LRP. Thus, the BiLRP provides a similarity model for the specific problem by verifiability and robustness.

The BiLRP is presented as a multiple LRP combined procedure and recombined on input layer. Here, x and x’ are input which are to be compared for similarity, $\emptyset_{x}$ as a group of network layer with {$\emptyset_{1}$ to $\emptyset_{L}\}$, and y(x, x’) as the combined output given in Equation (2).

(2) $$\mathrm{BiLRP}\left(y,x,x^{\prime }\right)=\overset{h}{\underset{m=1}{\sum }}\mathrm{LRP}\left({\left[\emptyset_{L}\circ \cdot \cdot \cdot \circ \emptyset_{1}\right]}_{m},x\right)⨂\mathrm{LRP}\left({\left[\emptyset_{L}\circ \cdot \cdot \cdot \circ \emptyset_{1}\right]}_{m},\;x^{\prime }\right)$$

The DeepLight LRP [17] performs decoding decision decomposition, which is used to analyze the dependencies between multiple factors on multiple levels of granularity. It is used to study the fine-grained temporo-spatial variability of the high dimension and low sample size structures.

#### 4.1.3. Statistical Functions for the Feature Analysis and Processing

The statistical analysis [21] of survivors and non-survivor’s comparison for categorical variables is performed by chi-square test/Fisher’s exact test and reported as interquartile range (IQRs) and standard deviation/medians. Whereas, the continuous variables by Mann–Whitney U test or Student’s *t*-test and expressed as frequencies. The Kaplan–Meier is used for graphical analysis of the relationship between two features with a significance log rank test. The hazard model of multivariate cox proportional regulation regulates the risk factor for the outcome and is analyzed graphically by the log-log prediction plot. In such cases, a significant *p*-value is less than 0.05 for single variate and 0.10 for bi-variate analysis. The generalized estimating equation (GEE) [23] is used to present the correlations between the feature matched sets. The incidence difference between the feature inheritance with GEE matching is within pre and post data adjustments. The Charlson comorbidity index score [25] is used to determine the comorbidities affected hospitalized patient life span risk within one year by a weighted index. The multivariate imputation is performed by the multiple imputation for the post-hoc sensitivity analysis for discrete and continuous data using chained equations. The lambda, mu, and sigma (LMS) method [29] is used to calculate the spirometric values for the normal lower limits in the z-scores. The kappa is an account chance agreement, where measurement agreement produces output as kappa 1.0 else 0. The least absolute shrinkage and selection operator (LASSO) [32] is a method of variable selection and regularization for improving prediction accuracy as a regression analysis. The imbalance classification problem is popularly solved by using Synthetic Minority Oversampling Technique (SMOTE) [44]. The cause of imbalance is usually due to the minority class, which are later duplicated in the training set before fitting the model. Such duplication helps to balance class duplication but does not provide any additional information.

#### 4.1.4. SHapley Additive exPlanations (SHAP)

The SHAP [35] uses ranking based algorithms for feature selection. The best feature is listed in the descending values by using SHAP scores. It is based on the features attribution magnitude and is an additive feature attribution method. SHAP is a framework that uses shapley values to explain any model’s output. This idea is a part of game theoretic approach which is known for its usability in optimal credit allocation. SHAP can compute well on the black box models as well as tree ensemble models. It is efficient to calculate SHAP values on optimized model classes but can suffer in equivalent settings of model-agnostic settings. Individual aggregated local SHAP values can also be used for global explanations due to their additive property. For deeper ML analysis such as fairness, model monitoring, and cohort analysis, SHAP can provide a better foundation.

#### 4.1.5. Attention Maps

Popularly used to be applied on the LSTM RNN model, which highlights the specific times when predictions are mostly influenced by the input variables and has a high interpretability degree for the users [51]. In short, the RNN’s predictive accuracy, disease state, decomposition for performance, and interpretability is improved. The attention vector learns feature weights, to relate the next model’s layer with certain features mostly used with LSTM for forwarding attention weights at the end of the network.

(3) $$a_{k}=softmax\;\left(W_{k}x_{k}\right)$$

Here, the $W_{k}$ learned weights are used for calculating $a_{k}$ for every *k* feature of $x_{k}.$ A feature on every time step is $x_{k}$ weighted with a learned attention vector, which is later given as $y_{k}$ in Equation (4).

(4) $$y_{k}=a_{k}⊙x_{k}$$

An ICU critical task to capture individual physiological data that is time sensitive is demonstrated in DeepSOFA [52]. The attention mechanism is used to highlight variables in time series, which are crucial for mortality prediction outcome. Successively, the time step is assigned with more weights thought to be more influential for outcome.

#### 4.1.6. Local Interpretable Model-Agnostic Explanations (LIME)

The LIME is a feature-scoring method, which performs the input data samples perturbation and checks for prediction change for understanding the model. In SurvLIME [53], cox proportional hazard is used to approximate a survival model within the range of the test area. The cox uses covariates coefficient of linear combination for the prediction impact of solving unconstrained optimization problems and other applications. The black-box-based human understandable explanations are given by medical examiner XAI [54], which is a LIME-like with rule-based XAI. In this case, a model agnostic technique is used for handling sequential, multi-labelled, ontology-linked data. This model trains a decision tree on labeled synthetic neighbors and the decision rules help to extract the explanations. The applications are used to predict the next diagnosis visit of the patient based on EHR data using RNN. The Lime based super-pixel generation is given in Appendix A.1.

### 4.2. Knowledge Base and Distillation Algorithms

#### 4.2.1. Convolutional/Deep/Recurrent Neural Networks (CNN/DNN/RNN)

CNN is a deep learning method, which is used to depict the human brain for higher performance and solving of complex tasks. It basically takes an input data/image, assigns weights and biases to its various factors, and later differentiates them from each other. The filters used here act as a relevant converter for spatial and temporal dependencies. The CNNs designed for structured output are used for image captioning [11]. To improve this captioning, the local discriminative image regions are found to be better with the CNN + LSTM models. The CNN scoring [12] provides precise localization. Later, based on some categories and thresholds, the scores are calculated. The DNN [43] is termed on the network consisting of multiple hidden layers. The DNN, once trained, can provide better performance for the suspicious image findings, which can be used to identify faults and status. The RNN is mostly used in the natural language processing applications as they are sequential data algorithms. It is usually preferred for remembering its input by its internal memory structure and thus is mostly suitable for machine learning methods involving sequential data. The bi-directional RNN [14] is designed to function as an encoder and decoder, which emulates searching through sequences at the time of its decoding. Thus, the sequences of forward and backward hidden states can be accessed.

#### 4.2.2. Long Short-Term Memory (LSTM)

The advancement for processing, classifying and making predictions on time series data is achieved by using LSTM. The vanishing gradient problem is popularly solved by using LSTM. The bi-directional LSTM [17] is used to model the within and across multiple structures with the spatial dependencies. Deeplight also uses a bi-directional LSTM, which contains a pair of independent LSTM iterating in the reverse order and later forwarding their output to the fully-connected softmax output layer. The LSTM encoder takes n-sized embedded sequences with dual layer, n cells, and outputs dense layers. The second LSTM is the reverse architecture known as a decoder to reconstruct the input. The dropout layer can be used in between encoder and decoder to avoid overfitting.

In this LRP, the linear/non-linear classifier f is used with input a having dimension d, positive prediction f(a) > 0, and $R_{d}$ is having a single dimension of relevance.

(5) $$f\left(a\right)\approx \sum_{d=1}^{D}R_{d}$$

Here, $R_{j}^{\left(l\right)}$ with a j neuron at l network layer, $R_{i\leftarrow j}^{\left(l-1,l\right)}$ defined by deep light where $Z_{ij}=a_{i}^{\left(l-1\right)}w_{ij}^{\left(l-1,l\right)}$ having coefficient weight w, a as input, and ϵ as stabilizer given in Equation (6).

$$R_{j}^{\left(l\right)}=\underset{i\epsilon \left(l\right)}{\sum }R_{i\leftarrow j}^{\left(l-1,l\right)}$$

(6) $$R_{i\leftarrow j}^{\left(l-1,l\right)}=\frac{Z_{ij}}{Z_{j}+\in .\mathrm{sign}\left(Z_{j}\right)}R_{j}^{\left(l\right)}$$

#### 4.2.3. Recent Machine Learning-Based Approaches

The support vector machines (SVMs) are used for regression, classification, and outlier detection, which are supervised learning algorithms. It is more popularly used in high-dimensional spaces, which can be even greater than sample size. The linear SVM [29] is used in ultra large datasets for solving multiclass classification problems, which is the version of the cutting plane algorithm. The polynomial SVM is also known as polynomial kernel, which shows the polynomial having feature space with a training set focusing on the similarity vectors. The decision boundary flexibility is controlled by degree parameter. Hence, the decision boundary can increase based on the higher degree kernel. The SVM also uses one more kernel function known as Gaussian RBF (Radial Basis Function). The value calculated on the basis of some point or origin distance is RBF kernel. In machine learning, a deep neural network class or generative graphical model is known as deep belief network (DBN) [31]. It is constructed with latent variables of multiple layers having interconnected layers excepts for the units in each layer. The deep rule forest (DRF) [39] are multilayer tree models, which uses rules as the combination of features to outcome interaction. The DRF are based on the random forest and deep learning based algorithms for identifying interactions. Validation errors can be effectively reduced by DRFs hyper-parameters fine tuning.

The DBN [55] consists of the following evolution of a restricted boltzmann machine (RBM) having posterior probability of each node with values 1 or 0.

(7) $$P\left(h_{i}=1|v\right)=f\left(b_{i}=W_{i}v\right)$$

(8) $$P\left(h_{i}=1|h\right)=f\left(a_{i}=W_{i}h\right)$$

Here, the $f\left(x\right)=1/\left(1+e^{-x}\right)$, which has energy and distribution function as:

(9) $$E\left(v,h\right)=-\sum_{i\in v}a_{i}v_{i}-\sum_{j\in h}b_{j}h_{j}-\sum_{i,j}v_{i}h_{j}w_{ij}$$

(10) $$p\left(v,h\right)=\frac{1}{z}e^{-E\left(v,h\right)}$$

The RBM follows unsupervised learning with pdf p(v), θ ϵ {W,a,b} as likelihood function, and v as input vector given as p(v,θ), where the gradient method has logp(v,θ) as likelihood function and higher learning can be achieved by revising gradient parameters as $\frac{\partial p\left(v,\theta \right)}{\partial \theta }$.

$$\theta \left(n+1\right)=\theta \left(n\right)+a\times \left(-\frac{\partial p\left(v,\theta \right)}{\partial \theta }\right),\;\;\theta \;\epsilon \;\left\{W,a,b\right\}$$

$$-\frac{\partial logp\left(v,w_{ij}\right)}{\partial w_{ij}}=E_{v}\left[p\left(h_{i}|v\right)\times v_{j}\right]-v_{j}^{\left(i\right)}\times f\left(W_{i}\times v^{\left(i\right)}+b_{i}\right)$$

$$-\frac{\partial logp\left(v,b_{i}\right)}{\partial b_{i}}=E_{v}\left[p\left(h_{i}|v\right)\times v_{j}\right]-f\left(W_{i}\times v^{\left(i\right)}\right)$$

(11) $$-\frac{\partial logp\left(v,a_{j}\right)}{\partial a_{i}}=E_{v}\left[p\left(h_{i}|v\right)\times v_{j}\right]-v_{j}^{\left(i\right)}$$

#### 4.2.4. Rule-Based Systems and Fuzzy Systems

A rule-based system uses knowledge representation rules for obtaining the knowledge coded in systems. They are completely dependent on the expert systems, which solves the knowledge-intensive problem by reasoning similar to human experts. It is used in stroke prediction models by interpretable classifiers using Bayesian analysis [56]. The interpretability of decision statements is simplified by the high dimensional and multivariate feature space by the discretization of if-then conditions. The decision list has posterior distribution yielded by Bayesian rule list. The structure used here to support sparsity has a highly accurate medical scoring system. The interpretable mimic learning uses gradient boosting trees and has high prediction performance as a knowledge distillation approach [57]. Mimic learning uses a teacher and student model, where the teacher model eliminates training data noise/error and soft labels are passed to the student model as regularization to avoid overfitting. It is applied in the medical domain of acute lung injury and achieves high prediction results. It is also known to be applicable in speech processing, multitask learning, and reinforcement learning. Fuzzy rules are a form of if-then conditional statements that are yielding truth to a certain degree instead of complete true/false. A deep rule-based fuzzy system is used to predict ICU patient’s mortality which consists of a heterogeneous dataset combining categorical and numeric attributes in hierarchical manner [58]. The interpretable fuzzy rules can be found in each unit of hidden layer within this model. Also to gain interpretability, a supervised random attribute shift is added in the stack approach.

The supervised clustering has fuzzy partition matrix and cluster centers. Here, $\beta_{dp}$ is the output weight vectors having a building unit as dp-th, where the partition matrix is $U_{dp}$ and output set as T given in Equation (12).

(12) $$\beta_{dp}={\left(\frac{1}{Const}I+U_{dp}^{T}\;U_{dp}\right)}^{-1}U_{dp}T$$

The interpretability is the layer’s prediction with random projections for higher linear separability, where $\alpha^{\prime }$ is the sub constants of α, $Z_{dp}$ as random projection matrix, and $Y_{dp}$ as the last unit’s output vector.

$$X_{dp}=X+\alpha^{\prime }Y_{dp}Z_{dp}$$

(13) $$Y_{dp}=U_{dp}\beta_{dp}$$

#### 4.2.5. Additional XAI Methods for Plots, Expectations, and Explanations

The partial dependence plot (PDP) in machine learning presents a marginal effect between input of one or multiple features on the final prediction, which is usually having a partial dependency. The PDP algorithm performs the average of all input variables except for PDP computed variable n [59]. This variable n is then checked in relation to the change in target variable for the purpose of recording and plotting. In comparison to the PDP, individual conditional expectations focus on specific instances that disclose variations in the recovery of the patient’s subgroup [60]. The XAI-based explanation to the classifier prediction is best achieved by the Local Interpretable Model-agnostic Explanations (LIME) as an interpretable model approximating black box model to the instance under consideration [61]. The artifacts are user defined interpretable modules and are used later to generate local black boxes for instance neighbors. The user intervention and artifact limits are overcome by Semantic LIME (S-LIME) for possessing semantic features which are independently generated using unsupervised learning.

The fidelity function is given below consisting of model g with the instance x and y for feature characterizing agreement and the function π having exponential kernel with weighted **σ** with a distance D.

(14) $$ℱ\left(x,f,g,\pi \right)=\sum_{y\epsilon X}\pi \left(x,y\right).{\left(f\left(y\right)-g\left(y\right)\right)}^{2}$$

(15) $$D\left(x,y\right)=\sum_{x_{i}=1}\left|x_{i}-y_{i}\right|$$

LIME is popular to highlight the important features and provides explanation based on its coefficient but suffers due to randomness in sampling step, making it unacceptable in medical applications. To gain trust, safeguard stakes, and avoid legal issues, a high stability and an acceptable adherence level system is proposed known as optimized LIME explanations (OptiLIME) for diagnostics [62]. The mathematical properties are clearly highlighted and are kept stable across several runs in OptiLIME to search for the best kernel width in an automated way. As per the formula given below in Equation (16), the declining $R^{2}$ is converted into $l\left(kw,{\tilde{R}}^{2}\right)$ a global maximum to get the best width. Here, the ${\tilde{R}}^{2}$ is the expected adherence with random kw values.

(16) $$l\left(kw,{\tilde{R}}^{2}\right)=\{\begin{array}{c} R^{2}\left(\mathrm{kw}\right),\;\;\;\;\;\;\;\;\;\;\;\;\;\;\;\;\;if\;R^{2}\left(\mathrm{kw}\right)\leq {\tilde{R}}^{2}\\ 2{\tilde{R}}^{2}-R^{2}\left(\mathrm{kw}\right),\;\;\;if\;R^{2}\left(\mathrm{kw}\right)>{\tilde{R}}^{2} \end{array}$$

In the classical ROC plot and AUC, the alterable threshold leads to the changes in false positive and false negative errors types [63]. As the partial part of ROC and AUC are useful in imbalanced data, then optional methods include partial AUC and the area under precision recall (PR) curve but are still insufficient to be trusted completely. Therefore, a new method known as partial AUC (*pAUC*) and c statistics of ROC are present, maintaining characteristics of AUC which are continuous and discrete measures, respectively. For the horizontal partial AUC, where x = 1 for the AUC integration border and other parts as true negative. Integration with baseline as *x*-axis and baseline x = 0 in case of swapping x and y axis. Thus, by transforming x (FPR) to 1 − x (TNR) then TNR can be received as required and x = 0 changes to 1.

(17) $$pAUC_{x}≜\int_{y_{1}}^{y_{2}}1-r^{-1}\left(y\right){\mathrm{dy}}^{}$$

The partial c statistic ($c_{\Delta }$) for ROC data is given in the normalized form as below in Equation (18). The $c_{\Delta }$ can be expressed as *J* out of positive’s *P* and the k as a subset out of negative’s *N*.

(18) $${\hat{C}}_{\Delta }≜\frac{2\mathrm{PN}.c_{\Delta }}{J.\;N+K.P}$$

The partial c statistic can be summed up as shown by the whole curve having q disjoint partial curves.

(19) $$c=\sum_{i=1}^{q}{\left(c_{\Delta }\right)}_{i}$$

### 4.3. Interpretable Machine Learning (IML)

Machine learning has made phenomenal progress recently in a wide variety of applications including movie recommendation, language translation, speech recognition, self-driving cars, etc. [64,65]. IML aims to provide human-friendly explanations with the combined efforts from computer science, social science, and human–computer interaction. As self-driving cars need to make decisions by themselves in real time, the black box model would not be feasible and acceptable. Therefore, an open box model with explainability will convey the decision to the user and its choice based on the related reason [66], i.e., why the daily route was changed is due to the traffic congestion in the upcoming lane. The two categories of IML can be given as:

#### 4.3.1. Intrinsic Interpretability

Inherently interpretable models consist of self-explanatory features within their structure. It has more accurate explanations with the slight trade-off of prediction performance. The global interpretable models can be either made by interpretable constraint or by complex model extraction. In interpretability constraints, the pruning of decision trees is performed to replace subtrees with leaves for deep trees instead of balanced structure. In the case of interpretable CNN, natural objects are identified accurately by adding regularization loss for learning disentangled representations, whereas in interpretable model extraction, also known as mimic learning, the trade-off of explanation is not substantial. In this case, the complex model is converted into a simple interpretable models, i.e., decision trees, linear model. The obtained model has better prediction and explainability performance, e.g., the ensemble tree or DNN is transformed into a decision tree, where the overfitting is handled by active learning.

The local interpretable models are more focused on providing specific prediction by a more justified model architecture. The attention mechanisms used by the RNNs as sequential models are interpreted by the attention weight matrix for explaining individual predictions. Attention mechanism is mostly used in image captioning with CNN for image vectorization and RNN for descriptions. Additionally, it can also be used in neural machine translation applications.

#### 4.3.2. Post-Hoc Interpretability

These are the independent model, which requires supporting models to provide explanation. The post-hoc global explanation consists of machine learning models that capture several patterns from the training data and retain knowledge into the model. Here, the knowledge within the pre-trained models are presented to the end user understanding. In machine learning, the data is converted to features, which are interpretable and are mapped to output, i.e., feature importance. Model agnostic explanations are known to be a black box model with no transparency, whereas in permutation feature importance, the n features are shuffled to check the model’s average prediction score and is known to be an efficient and robust strategy. The model-specific explanation is based on internal model structure for its explanation. The generalized linear models (GLM) consist of linear model combinations for features transformation, e.g., linear regression, logistic regression, etc. GLM has limitations when the feature dimensions become too large. In tree-based ensemble models, i.e., random forests, gradient-boosting machines (GBM), XGBoost, which measure feature contribution by accuracy, feature coverage, or split data count. In case of DNN explanation, the representations are given by the neurons at the intermediate layers for detail analysis. The activation maximization is utilized for iterative optimization of the image interpretations at different layers. Even though some noise and errors can be faced during classification, generative models are found to provide better visualization. Therefore, the CNN can capture better visualization from object corners, textures to object parts, and then whole objects or scenes. The RNN are better known for abstract knowledge where language modeling is required for learning representations. The RNN are good at capturing complex characteristics such as dependencies, syntax, and semantics. RNNs can capture hierarchical representations from different hidden layers, whereas the multi-layer LSTM are used to construct bi-directional language models with context aware understanding of words.

The post-hoc local explanations are focused on individual predictions based on the features supporting it and are also known as attribution methods. In model-agnostic explanations, the predictions from different machine learning models as black boxes are explained without guarantee, whereas the local approximations explanation supports interpretable white box based explanation in an adjacent part of the input, e.g., attribution methods, sparse linear models such as LASSO. The perturbation-based methods, the feature contribution, determines the prediction score. Thus, if the input part can change the prediction, then it is known as counterfactual explanation. The model specific explanations refer to white-boxes such as back-propagation method, deep representations, and perturbation methods. For perceptive interpretability, refer to Appendix A.2.

## 5. Characteristics of Explainable AI in Healthcare

**Figure 4:**
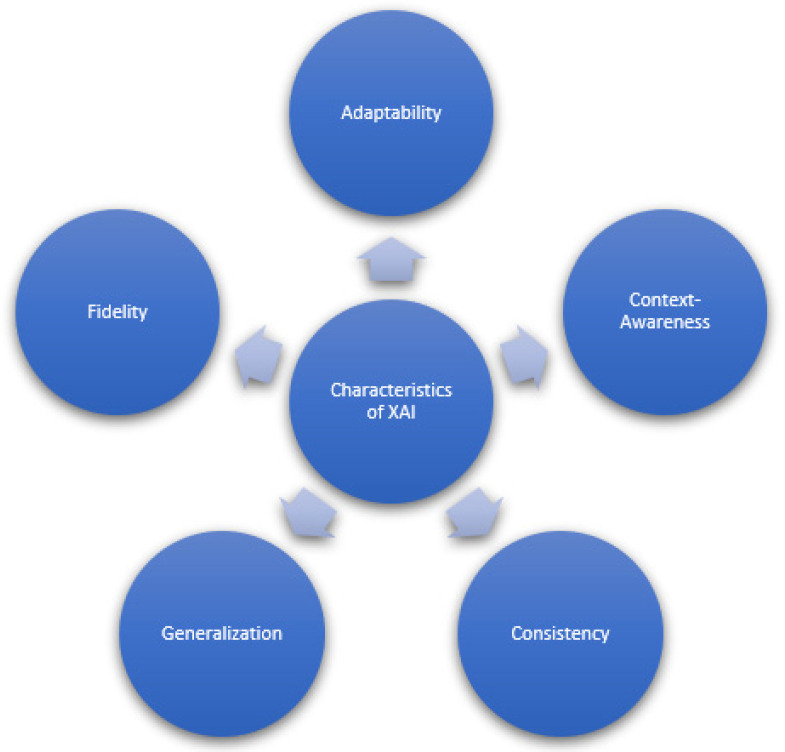
XAI Characteristics.

In this section, a complete aspect of the medical XAI system is given in detail. Considering the hospital situation, the interaction, explanation, and transparency detail of the system will be disclosed. The characteristics will provide a complete overview about the new generation of XAI healthcare system, equipped with enhanced capabilities [66] as shown in Figure 4.

### 5.1. Adaptability

The transparency needs to be provided equally amongst all the healthcare system users. In case of medical examiners, the details of complete dataset preprocessing, algorithm function and decision analysis at each step should be provided. The medical examiners should be familiar with the system usage by training provided earlier to consultation and the protocol followed by the hospital treatment standards. The detailed decisions based on the model training on the previous year’s consultations can help the medical examiners to check on multiple factors and then provide a final decision.

Early prediction systems can help medical examiners to take immediate actions to avoid severe conditions. In case of nurses, the statistics of the patient’s health can be displayed to help them record the patient’s health recovery and administer the required procedure. The history records of the patients should be accessible and should provide reminders about the emergency and regular scheduled procedure to be achieved. For the administrators, the patient’s record, clinical tests, previous history of payments, and alerts for the future treatment possibility as decided by the medical examiners can be predicted. The patients connected to the hospital system can receive the daily reminders of the personal treatment, doses, warnings about the diets, alerts for the improvements and updates from the system in case of major infections spread, etc.

### 5.2. Context-Awareness

An XAI system should be complete in every sense. In case of diagnosis, the system should provide detailed vital signs, CXR, clinical tests are given as the affected patient’s conditions and disease. Hence, a prediction or classification system is used to provide the discharge status after one week or month based on the patient’s history records. For the surgical/ICU department, the features would vary as the oxygenation, ventilators, supporting instruments based on the alternative mechanical methods, etc. Therefore, the algorithms used in this XAI model should be adaptable to the new feature depending on the patient’s case-to-case basis. In the drug development/consultation process, the XAI algorithm can predict the required dose schedule, the weighted contents of the drug, the combination of drug should be suggested for the comorbidities case, etc. The risk associated with the different types of cases should also be disclosed in the drug usage case. Exceptional circumstances can also be made for high risk patients and the supporting dietary or treatment with different age and continental patients should be constituted.

### 5.3. Consistency

The healthcare dataset and model should be consistent during the continuing patient’s treatment. Also there should be consistency between multiple evaluations for the same patient. Therefore, versioning is required to be maintained and report the updates as per the module changes. A version reports the updates as per the module enhancements. A version report can be made available to know the updated module details and the changes affecting the past patient’s records. Log records should be maintained for every patient that can display complete history with health status and respective time series records. The system log records should be immutable and must store the versioning information with the updates and fixes. A database maintained with such rules must also include the patient’s medication course applied, clinical test report, ICU/emergency facilities details and some special treatment applied based on some exceptional circumstances. The comorbidities are related to complex cases that may require careful treatment and dependency factors to be analyzed. Consistency is an important aspect of the hospital’s quality control and research department.

### 5.4. Generalization

In the healthcare system, every patient’s data consists of vital signs, CXR, clinical tests and comorbidities. In case of ICU treatment, additional features are present. The designed model must be able to distinguish between multiple patients based on the features with high accuracy and less error rate. Thus, if many instances have similar explanations, then the generalization is not acceptable for the treatment and operating process. The XAI model should be adaptable to different features and must be effective to provide distinct explanations based on the case-to-case basis. The XAI algorithm must be able to provide high transparency of every category of the patient’s data, i.e., vital signs, CXR, clinical test, and comorbidities. It will be useful to distinguish between patients’ affected status in different categories. These explanations will be helpful to the medical examiners and medical staff for knowing about the patient’s current health status, i.e., slight/mild/severely affected and to take appropriate further actions.

### 5.5. Fidelity

A designed XAI model should be configured as per the available dataset categories and must be specific to the objective application, i.e., healthcare. To provide a more effective explanation, the model must be interpretable. Thus, the benefit of having interpretable models is to analyze the processing of the input data at each level. Considering the CXR images, the interpretable model will provide analysis by CXR image quality as high, average, and low. Additionally, to know whether the CXR processing for the chest cage edge identification is aligned or not. The feature identification for the different categories of diseases/infection as infiltrate, cardiomegaly, effusion, COVID-19, etc. The level of severity analysis of the patient’s condition either as normal, slight, mild, and severe infection are some of the factors. Furthermore, interpretation must be aligned with the XAI model prediction to enable the patient’s discharge and/or mortality status prediction with high transparency.

## 6. Future of Explainability in Healthcare

**Figure 5:**
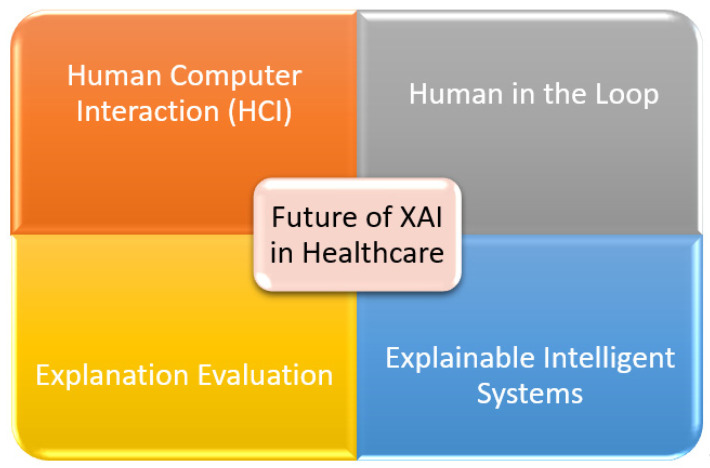
XAI in Healthcare.

In this section, we have identified and provided the four key aspects for the future of explainability in healthcare. The human-in-the-loop (HITL) enhances the classification capability of XAI, human–computer interaction (HCI) provides the deep understanding of a patient’s condition, explanation evaluation provides key insights for personalized outcome, and explainable intelligent systems (EIS) significantly improves the medical XAI system. The demands of the medical system in healthcare are always at priority. The future of XAI shows promising solutions that can improve the healthcare facilities as shown in Figure 5.

### 6.1. Human–Computer Interaction (HCI)

The concept of HCI refers to the interaction between the real world and augmented reality [67]. The human subject here is the patient whose interaction with the computer is recorded for the symptoms feature identification purposes. Here, the computer sensors are used to record the human movements, e.g., sneezing, coughing, chest pain, stress level, lack of focus, etc. The HCI then provides the output based on machine learning algorithms for the predictions of the results. The HCI is also a crucial aspect in the future of XAI as it will add the symptoms feature for disease identification. The HCI has further applications to detect human poses, body structure, movement discontinuities, speech recognition, object handling using motion detection, psychological response, etc. Even though the recent AI is thought to be progressing, with the future XAI, a complete human body functioning is thought to be a progressive step towards the goal.

### 6.2. Human in the Loop

Applying the XAI concept in the healthcare domain is thought to be incomplete without the human in the loop process [68]. Considering an infection/disease, there can be several symptoms including EHR, CXR, clinical tests, etc. In recent works, it can be noticed that multimodal data analysis is a challenge for the machine learning algorithms because of trade-offs, less domain knowledge, high false positives, etc. To effectively solve such a challenge, the domain expert should be continuously involved within the interpretable model implementation to set the required hyper-parameters at each level, manage the trade-off, add/remove features manually, decision-based system, manual labeling of data, handling exceptional conditions, etc. A versioning-based system or feedback evaluation system should be used for continuous improvement so that the final system will be used in the hospital evaluation with trust. Human in the loop is hence necessary to manage the identification/diagnosis or prediction system for the new category of infection/diseases without replacing the whole XAI model and by adapting to the current scenario.

### 6.3. Explanation Evaluation

The XAI explanation for the final results evaluation is one of the most crucial aspects in healthcare. During the peak hours for patient’s diagnosis and health prediction, medical examiners prefer to only check the final result as an expert opinion. Therefore, the final explanation provided by the system should be effective and acceptable. Nevertheless, recent works have discussed the selection of the explanation from multiple robots [69]. For the different robots the explanation may vary, so during the initial phase of the model deployment in the hospital center, the medical examiners are asked to choose the sentence type from the multiple explanation options as best suitable to the respective medical examiner/user. The type of explanation selection determines which robot is most suitable to the medical examiner and is thus finalized to that specific medical examiner’s personal account. Therefore, both the system transparency of the evaluation and the explainability are achieved. The detailed explanation of the results provides model interpretability and helps to gain the user’s trust.

### 6.4. Explainable Intelligent Systems

Modern healthcare is being strengthened and revolutionized by the development in AI [70]. The XAI-based system can improvise the previous analysis, learning, predict, and perform actions with explainability for the surgery-assisted robots, relationship within genetic codes to detect, and evaluate minor patterns. The XAI intelligent system is aimed at explaining the AI-led drug discovery, so that faster, cheaper, and effective drug development is performed, e.g., COVID-19, cancer treatments, etc. Healthcare robotics are used for assisting certain patients in paralysis, smart prosthesis, assistive limbs, spinal cord injuries and can explain how much recovery in the patient is recorded. Additionally, during the surgery process, the robots can explain the decision taken and necessary actions. The AI-powered stethoscope can be used in remote areas where medical personnel shortage is present and can analyze high clinical data for discovering disease patterns and abnormalities. Ultimately, the intelligent systems can treat and provide better explanations for transparent and trustable processes.

## 7. Prerequisite for the AI and XAI Explainability

**Figure 6:**
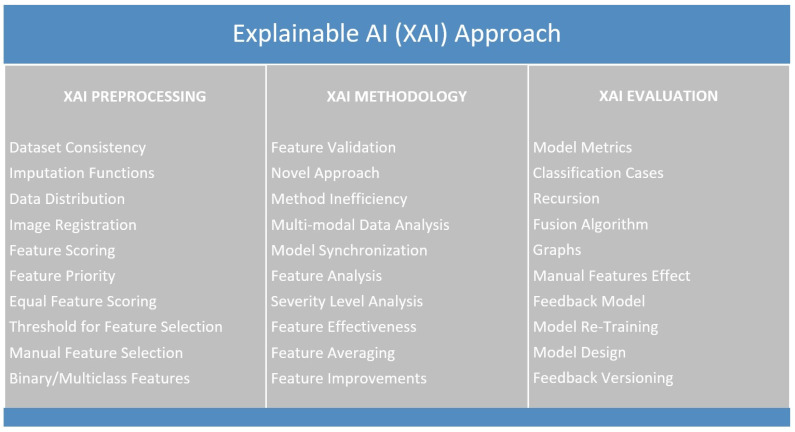
Explainable AI Approach Planning.

A user is recommended to choose complete XAI explainability categories of preprocessing, methodology and healthcare as shown in Figure 6, as a part of the human-in-the-loop approach with the discussions provided in the following subsections:

### 7.1. Discussion for the Initial Preprocessing

▪ Whether the dataset is consistent?

The dataset is the input given to the model for its processing. In practical aspects, the dataset is not always complete, as it may include missing data, incomplete data, etc. Thus, consistency within the dataset is very crucial. Therefore, the dataset should always need to be checked prior to the utilization, as it may lead to miscalculation for predictions.

▪ Which data imputation functions are required for data consistency?

In case of an inconsistent dataset, which is usually encountered by the researchers, an appropriate selection of data imputation techniques is quite necessary. This process can also be known as cleaning, which performs fixing inaccurate values by deleting, modifying, or replacing the records. The imputation operations include missing/non-missing at random, mean, median, replace by zero or constant, multivariate imputation by chained equation (MICE), stochastic regression, interpolation/extrapolation, hot deck, data augmentation, etc.

▪ Presentation of analysis of the data distributions?

The dataset can be analyzed by its distribution in detail. The distribution is used to present the relationship between observations within the sample space. There can be various types of distribution, i.e., normal, uniform, exponential, Bernoulli, binomial, poisson, etc. The distribution will provide an idea by the analysis with the graphical presentation.

▪ Image registration techniques required for the image dataset?

In medical image processing, the input given for the chest X-rays (CXR) is not always consistent. For the alignment, the scene/object must be aligned in the correct angle. Thus, the issues of image scaling, rotation and skew needs are addressed by using image translation.

▪ Whether some feature scoring techniques are used prior?

Recently a need for high accuracy and productivity is present within the medical domain. Thus, feature engineering and scoring helps us to achieve this goal. The feature scoring is calculated based on the relevant features obtained by local explanation which has optimal credit allocation using SHAP. Several other methods include GRAD-CAM, saliency maps, LRP, Deep LIFT, LIME, etc.

▪ Is there any priority assigned to some features by domain experts?

In case, the expert will be supportive for achieving better prediction accuracy.

▪ What actions are taken in case of equal feature scores?

In the complex cases of feature scores showing equal values, the domain experts have to take the decision as to which features need to be considered on priority. There can be top 20 or 30 features shown by the feature scoring techniques, but the least important features having high variability needs to be eliminated. In such a case, manual selection of features on a case by case basis would be applicable.

▪ Is there some threshold assigned for feature selection?

Possessing several features is usually not effective, as it may lead to high imbalance within the dataset. Thus, applying thresholds to sort features as priorities can also be thoughtful to better prediction. In case of general ward patients, the thresholds applied are on the age, pulse rate, body temperature, respiratory rate, oxygenation (SaO2), etc. are considered to be beneficial.

▪ Are the features selected based on domain expert’s choice?

Comorbidities may cause complications in some rare patient cases. To handle such a situation, the domain expert/medical examiner can select a set of features from a particular sub-category including the ICU features. The categories of severe patient or critically ill can be given as slight, mild, or severe.

▪ How are binary or multiclass output based features used?

There can be binary or multi-class based output that can be managed effectively to provide considerate prediction. The domain expert in binary case can select either a class 0 or class 1 for priority, whereas for multiclass, a specific priority listing can be assigned to the features with that multi-class features.

### 7.2. Discussion for the Methodology Applicability in XAI

- What feature aspects make the method selection valid?

The machine learning algorithms are divided into multiple categories, i.e., unsupervised/supervised, regression, clustering, classification, etc. For a small dataset, principal component analysis, singular value decomposition, k-means, etc. can be applied. In the case of a large dataset, where speed and accuracy is important then classification algorithms experimented are SVM, random forest, XGBoost, neural networks, etc.

- Is the approach genuine for the system model?

A good survey paper reference will be useful to know the recent models and their respective results. Therefore, selecting an appropriate method for the preprocessing for feature scoring then using a suitable algorithm based on the available dataset by performing multiple experiments based on the shortlisted/recent models with hyper-tuning can yield better results. A good sense of data behavior will be useful for selecting the suitable model and configuring neural network architecture with parameters.

- Why would some methods be inefficient? Are references always useful for literature?

A recent literature works before or during the initial work of the XAI-based project would be crucial in this case as given in Table 1, Table 2, Table 3 and Table 4. It is recommended that instead of implementing all the methods, a reference from several books and papers can save time and help to understand different model behavior based on the dataset availability, thus helping us to know which methods can be better from the survey paper.

- In case of multi-modal data analysis is the model suitable? Will it be efficient to use such a model?

A multi-modal dataset includes data from different categories/formats (i.e., raw data, images, speech) required to be evaluated by a machine learning/artificial intelligence model with a binary or multiclass output. Such multiple data is hard to be evaluated by a single model. Thus, a hybrid model consisting of a combined independent model is made to ensure appropriate processing for the respective format data, which is later combined by regression, voting, ensemble, classification predictions, etc. Appropriate combination methods used will have efficient performance.

- How is the model synchronization made for input?

Multi-modal data has different input provided to the respective same/different models. A separate algorithm is present, which collects the output of both the models and gives the prediction/classification. Thus, the synchronization is achieved in this process in parallel.

- Are the features of the current patient affected more or less than the average dataset?

The processing model must provide the details of the patient’s condition by his features. During the prediction of the results, it is expected prior by the XAI to provide the patient’s condition in detail comparison to the population for the domain expert analysis and acceptance with trust.

- What is the current patient’s stage the methods have classified?

The XAI informs in advance about the patient’s affected stages, i.e., stage 1, stage 2, and stage 3. These disease-affected stages depict the critical condition the patient is at present. The patient affected stage is useful for the medical examiner to provide the required medical treatment.

▪ What is the current patient’s medication course assigned and its results?

Upon assigning a medication course, the medical examiner can check the patient’s recovery progress and can change/update it accordingly. In case of comorbidities, the patient medication course may vary. A medication may have different recovery progress based on case to case basis.

▪ How much of a percentage of a patient’s clinical features are affected?

An affected patient’s data such as vital signs, clinical laboratory features, intensive care unit (ICU) features, and medication courses are crucial for the complete status overview. The overall recovery of the patient can be expressed in percentage, which must provide detailed patient’s features for the confirmation.

▪ Are the features showing positive or negative improvement status?

With the hospital admission, a medical course in the standard operating procedure (SOP) improves the patient’s condition which shows a positive improvement. In rare cases of speciality treatment requirements, negative improvements can also be seen in the patient’s status. Thus, the patients are required to be shifted to the specialty care to the different section or hospital ICU.

- Which output metrics are suitable for the model evaluation?

A learning model is evaluated either on a statistical function, machine learning, or AI. The statistical function usage provides numerical *p*-value or graphical results, whereas machine learning models provide prediction with the metric of accuracy and deep learning by classification. In fact, all the metrics are suitable but the medical examiner can select the one which is more accurate and easy to interpret.

### 7.3. Discussion for the Evaluation Factors in XAI

- What cases are important for the output classification?

In case of binary output, the learning model must provide the classification as either infected or not infected (Yes/No), whereas for multi-class output, the learning model must classify clearly about the infection, current stage and improving/deteriorating condition by handling the false alarms carefully. The XAI explainability in such a case will play a crucial role.

- Is the output improving based on recursion?

The AI expert/architect can design the model carefully with the necessary parameters or layer configuration. The model can either be back-propagation, epoch based or a feedback model. It is a best practice to update the training at regular intervals with auto-weights adjustments. In the case of a feedback model, the domain expert’s suggestions are considered for the feature engineering.

- How are the multi-model outputs combined?

As discussed previously, the multi-modal data handled by the multiple models are combined as approximated by an algorithm. The output can then be represented either by a profitability value/prediction/graph. The limitation for multiple model systems is to overcome for handling a single model output.

▪ How are the bar graphs compared and evaluated?

The bar graphs are usually drawn by the iteration value and its respective prediction. The graphs are given for AUROC curve, PR curve, sensitivity vs. specificity, NPV, PPV, etc. The graphs are crucial in any system for the performance analysis as well as its effectiveness.

- Whether the user/domain expert likes to manually select features for evaluation?

In some special cases of comorbidities, there can be many false positive alarms that can cause panic. To handle such a situation, the domain expert can select manually a group of features by his choice and can take an appropriate treatment decision for the affected patient’s ahead.

- Is the system designed to record feedback from the domain experts?

As none of the system is considered perfect but it is supposed to continuously update itself for improvement. The feedback from the domain experts can resolve major issues about the new infections or its variants, which are not known by the trained system before and may compromise on performance.

- Whether the model updates training features with every evaluation?

The model re-training takes high processing time, which is a major issue in the ML/DL models. Therefore, an appropriate schedule is planned by the domain experts and research team to update the model based on the couple of weeks/months interval. Re-training is important for the system adaptation for the future tasks, and alternatively it can be done by proxy system for transferring weights later.

- Which model is suitable for such medical cases, machine learning (ML) or deep learning (DL)?

A white box model such as a decision tree is easy to understand but cannot be easily applied on complex human body features, as it may react differently than one another. Some ML models are known to work with high accuracy for some PPG disease cases, whereas for several other major infections/diseases, an interpretable deep learning-based model is required to provide explainability for every step of the multi-class output with the growth analysis.

▪ How the feedback suggestions by domain experts are adapted in the current model?

In case of data imbalance, the domain expert may decide to remove some features from the classification for high accuracy. However, if the feedback consists of adding scoring functions features, i.e., CCI, SOFA, OASIS, APS, SPSS, etc. then it is updated in the new version. The system adaptability is crucial for its progress.

Table 5 provides the problems addressed by the references as given for the Section 6, even though there exist some concepts that still need to be worked on by the future XAI research, including multi-modal data analysis [5], model synchronization, recursion, fusion algorithm, manual features effect, feedback model, model re-training [15], model design [15,16], and feedback design.

## 8. Case Study

**Figure 7:**
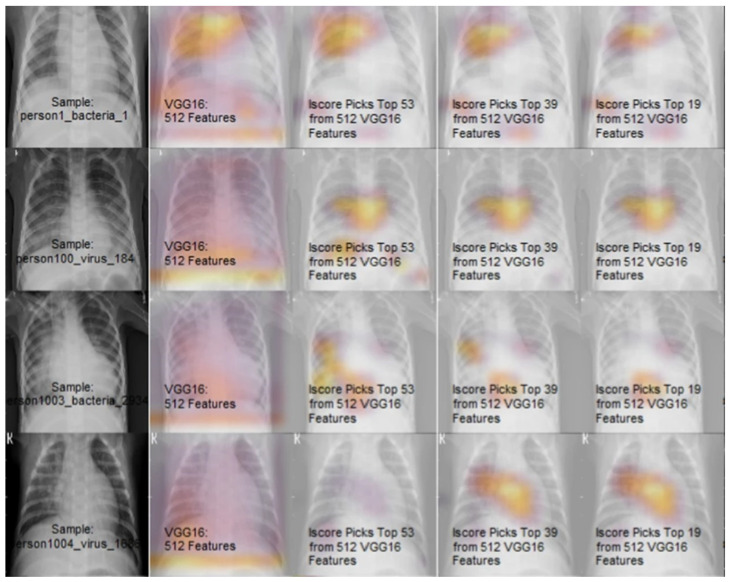
I-Score Model shown by reducing the top features.

The recent progress in XAI have further advanced the research with higher accuracy and explainability. Table 6 shows some of the medical datasets with references. The following discussion will help to understand the influential score (I-score) for the affected pneumonia patients [71] in Figure 7. The EHR data is known to possess many challenges, which would be very interesting when the supporting decisions taken for predictions are explainable. 

An interaction-based methodology is proposed for the image noise and non-informative variables elimination using I-score for feature prediction. The explainable and interpretable features are used to demonstrate its feature prediction, which has an interactive effect. Even though there is a tradeoff observed with the learning performance and effectiveness of explainability, it can still be overcome by providing new features scoring methods. An explanation factor is determined clearly by the prediction, which is related to the feature selection technique known to be interpretable with explainable. In this case, I-score variable used for explainability is processed in discrete form, which may be converted from the continuous variable as required. 

The largest marginal I-score is also specified as a random variable drawn from a normal distribution. The I-score is maximized by the optimal subsets of variables which are searched by the backward dropping algorithm (BDA). The BDA achieves its goal by variable elimination in a stepwise manner from an initial subset in a variable space. In this research, a pre-trained CNN is applied before I-score and/or alternative BDA, which is later evaluated using a feed-forward neural network requiring less number of parameters. The predicted 512 features from pneumonia affected CXR are further reduced to top 19 features which are explained to provide warning about the disease location.

The EHR used for the prediction of acute critical illness (ACI) in the hospitalized patients needs to be explained by an open box model [81]. The open box model is usually an interpretable neural network model with explainable AI (XAI). The drawbacks of AI models are known to lack correct results, i.e., identifying false positives or true negatives for critical care. To overcome this problem, the XAI-EWS is designed to provide visual explanations for sepsis, AKI and ACI. The architecture of XAI-EWS consists of deep Taylor decomposition (DTD) with temporal convolutional network (TCN) in the prediction module and a separate explanation module. The explanation module is used to support prediction relevant to the clinical parameters. These clinical parameters are listed in the form of top 10 parameters with high weights to recent values. The XAI-EWS model provides the user with the transparency of the system model and helps to earn trust by giving explanation of every key decision within the algorithm. The XAI-EWS has an individual and population based perspective for the model based explanation. The DTD has been beneficial for predicting the development of ACI from the individual perspective. Back-propagation is used for the relevance output processing with a global parameter having mean relevance scores and the correlations in the clinical parameters in local explanation of the population perspective.

The Influence score (I-score) and the backward dropping algorithm are used in combination for the Figure 7 output demonstration. This proposed methodology includes selecting high potential variables for influential modules, which is filtered by inter-activeness and later combined for the prediction. This I-score is known to work better with discrete variables. In case of random variables taken from normal distribution, then optimal cut-off is set to the highest marginal I-score. It supports limited categories to avoid classification error rates.

(20) $$I=\sum_{j\;\epsilon \;Ƥ}^{}n_{j}^{2}{\left({\bar{Y}}_{j}-\bar{Y}\right)}^{2}$$

Here, ${\bar{Y}}_{j}$ is the average of *Y* observations over the local average *j*th partition and global average Ƥ. *Y* is the response variable (binary 0/1) and all explanatory variables are discrete. ${Ƥ}_{K}$ is a subset of *K* explanatory variables {$x_{b1}\;\ldots \;x_{bk}\}$. $n_{1}\left(j\right)$ is the number of observations with *Y* = 1 in partition element j as given in Equation (20). The BDA algorithm is used as a greedy algorithm, which selects optimal subsets of variables having highest I-score. The architecture consists of an interaction based convolutional neural network (ICNN).

The explainable deep CNN (DCNN) is used for the classification of normal, virus infection (pneumonia), and COVID-19 pneumonia-infected patients [82]. A fine distinguishing criteria is set by designing application specific DCNN for different infection categories with high accuracy.

The training set consists of a gold-standard diagnosis set by a radiologist by confirmation. The training set provided is quite balanced in this system consisting of healthy, pneumonia-infected, other virus-infected and COVID-19-infected, thus providing high accuracy by avoiding trade-off of features within infection categories belonging to the same patients.

**Figure 8:**
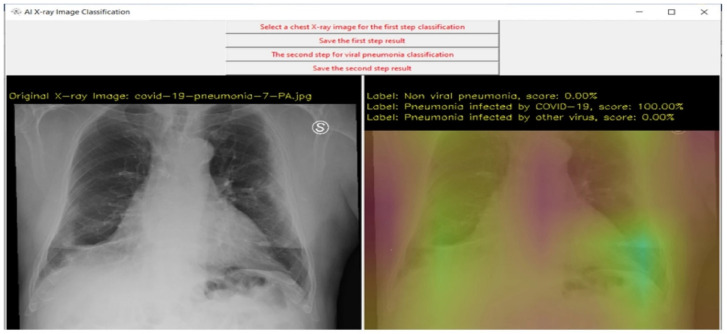
Explainable DCNN for CXR analysis and COVID-19 pneumonia classification.

The base model is adapted from VGG-19, where its convolution kernel is set as per the requirements. The final values are set with bright colors to identify the region of interest for medical analysis. The hyper-parameters are trained using the grid search to find the best settings. The two CNN models include CNN1 for training samples with category labels, test samples to partition the standard set, and CNN2 for the virus infection output. Finally, the CNN2 generates the output by classifying the infection in Figure 8 detail.

**Figure 9:**
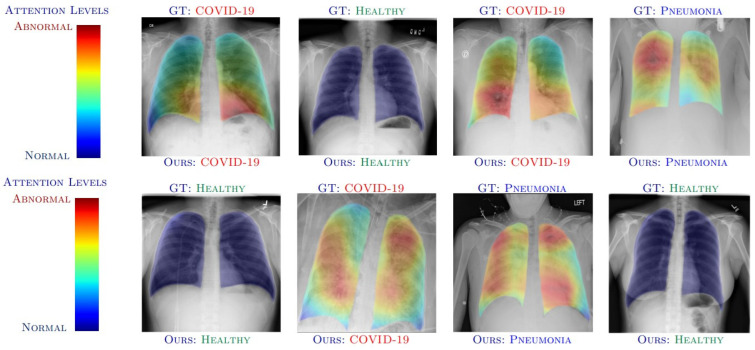
GraphXCovid pneumonia CXR results.

The graph diffusion pseudo-labelling by deep network for CXR-based COVID-19 identification is presented [83]. The Figure 9 GraphXCovid is used for COVID-19 identification by using a deep semi-supervised framework. It performs pseudo-labelling generation using dirichlet energy by a novel optimization model. Thus with minimal labels a high sensitivity in COVID-19 is generated. An iterative scheme in a deep net and attention maps are the highlights of this model. The work is considered to be the successor of deep SSL technique [84,85,86,87] combining generalization and feature extraction of deep neural networks. Therefore, the process can be given in detail as optimizing epochs for deep net extraction for graph construction, diffuse labelled sets to un-labelled data. Thus, pseudo-labels are generated, which optimizes the model parameter by regular updates, which is later iterated until completion. In this case, the medical data imbalance problem is handled during the diffusion process.

**Figure 10:**
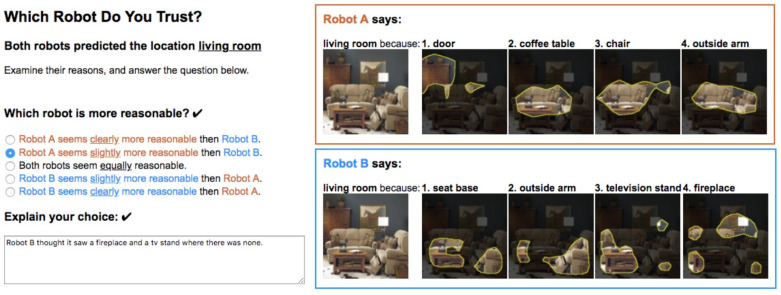
Feedback system for the human rating-based evaluation.

The feedback system is designed to evaluate which robot explanation is more suitable for an explainable AI system [69,88]. In the initial implementation which consists of having multiple feature evaluators such as CAM, Grad-CAM, and network dissection are used to support explanation by the robot as shown in Figure 10. The feature engineering pre-processing uses top 20% by the heat-maps, which are labeled with the respective concepts. The classification models then provide detailed accuracy for explanation by Resnet-18, Resnet-50, VGG 16, and AlexNet.

Even though the outcomes are the same by best accuracy, the explanations are distinct. In such a case the user can decide which robot explanation is more suitable for his understanding, and based on that the further outcomes are planned to be explained. The selection is taken from multiple questionnaires at the beginning, which are later evaluated based on five points Likert scale. In parallel, the suggestions given by the user in the feedback box are also collected for the advancements in the new version [89].

Nevertheless, multiple surveys for computer vision [90], deep learning [91], AI imaging techniques [92], and explainable AI [93] are present, which are informative and provide basic as well as in-depth knowledge about the concept and applications.

## 9. XAI Limitations

In order to claim the research work to be XAI-compatible, the designed model should be rated based on its explainability level and XAI evaluation. In practice, many XAI systems are not adaptable to the new challenges of model tuning and training [71]. Even though some models are well-designed but are not correctly trained and classified, which usually suffer from performance issues. Therefore, such challenges are hidden and are discussed in more detail as follows.

### 9.1. Explainability Ratings for the New Models

Several recent works claim to be XAI compliant but no specific rating based standard is present [71,82,83]. Therefore, at the base level a system can be checked for preprocessing, methods, and post evaluation explanation of the model which can still be further improved by point-based ratings. The explainability also depends on the ease of understanding and the prediction/classification of the XAI system usage. The language compatibility and parameter settings provide more transparency. The data statistics provided at every level and interpretability at every DNN layer can further improve the transparency with more explanation [94]. The decision transparency is rarely dealt with, and the user has no exact knowledge about the sudden change in decision [71]. Therefore, the decision taken at every step should be disclosed to the user, which can be helpful in the scenario of having false alarms and leading to chaos within the hospital staff.

### 9.2. Measurement System for the XAI Evaluation

During the XAI evaluation phase, the results comparison is not measurable [81]. Recently, robot-based explanations popularly known as bots have different classification explanation depending upon distinct models [88], i.e., CAM, GRAD-CAM, Network Dissection, etc. Subsequently, the CXR feature highlighting may differ by distinct models. Therefore, in such a case, the user’s profile account having different priorities can make the required settings for such preferences optionally by using multiple XAI analysis techniques known as intrinsic and post-hoc, which are used everywhere [83].

It is still unknown how to apply an automatically adapting system that can select different optimal techniques to provide the best classification/prediction. Ensemble algorithm can be one of the optional solutions but it uses a brute force method and leads to a performance trade-off. Nevertheless, a local dataset does tend to bias the classification and is not effective enough on the global data. A severe security threat for making the model training biased is made by adversarial attacks [95]. Therefore, identifying such a bias issue is also a challenge.

### 9.3. XAI System Adaptation to the Continuous Improvements

Many of the recent works have designed a specific hyper-tuned classification model that may have shortcomings/over-fitting on the different dataset or feature modification [82,83]. Thus, a model will not perform significantly for the transfer learning cases too. The XAI system must also be open to adapt to the new feature set, i.e., vitals signs, CXR image, ICU parameters, clinical tests, etc. [96]. Modification of such features by using a user feedback system is crucial for the system’s upgrade. A user feedback compatible system improves the model scope and is thought to be continuously adaptable. In case of model with input of single or multi-modal data source, the user must be able to choose either of the option as per data available, e.g., in a multi-modal system, if the CXR is not available for the patient’s disease diagnosis, then it must be able to classify on single source of vital sign or different data source of ICU parameters/clinical tests, whereas the model parameter tuning is also important for optimal performance, which needs to be performed automatically without sacrificing performance.

### 9.4. Human in the Loop Approach Compatibility

The saliency map analysis is not perfectly designed to identify certain features with vital signs data or CXR image data [71]. Therefore, the medical examiner’s based data labeling is required at the initial training phase of the model for better quality and to achieve high classification output [97]. Higher expectations from XAI has subsequently led to an intelligent system that can discuss and convince its classification to the medical examiners, so that an effective decision about the medical treatment can be made. Achieving such a highly capable human in the loop can greatly benefit the XAI progress. In addition to the previous discussion about user profile management for priority-based feature selection, XAI must be able to serve also in the multi-specialty hospitals concerning different departments. Interest for the single patient’s health analysis is one of the major challenges. Considering the XAI transparency and explainability at every DNN layer, the user must also be able to configure the layer size and weights for the effective analysis of diagnosis or severity analysis by feature highlighting is necessary.

## 10. XAI Recommendation System (XAI-RS)

Table 7 for XAI post-treatment recommendation system is beneficial to the hospitalized/treated patients for a group of diseases. As discussed earlier, the hospitalized patients were treated for a group of diseases i.e., pneumonia, bloodstream infections, acute kidney injury, mortality prediction, etc. have different features and symptoms. Therefore, the XAI-RS can evaluate the results as per the recent health condition of the discharged patient. For every patient, the XAI-RS will be personally evaluated. Thus, the patient’s recovered from AKI will have a default recommendation set with additional suggestions for personalized evaluation.

Table 8 presents the XAI-RS for the AKI-affected patient. These recommendations are default to every AKI-discharged patient but if the patient is addicted to smoking and/or consuming alcohol, then an additional recommendation needs to be added as shown by the orange highlighted box. The purpose of the XAI recommendation system is to provide best continuous treatment to the post-discharged patients to live a healthy lifestyle.

## 11. XAI Scoring System (XAI-SS)

The XAI-SS determines the standard grade for the newly designed and in use XAI systems. This scoring system in Table 9 can be extended to multiple areas, i.e., industrial, finance, sensor communications, etc. The scores can be assigned based on international (10 points), group (8 points), and local (6 points) policy achievements. As each of the 10 XAI factors are assigned equal scores with balanced/equal weightage, the final evaluation grade is assigned as Class I (≥90%), Class II (≥70% and <90%), and Class III (≥60% and <70%). Designing a new XAI system must involve experts for setting the objectives for a high quality work plan.

The training provided to the XAI system must involve an international dataset for its effectiveness (Table 6), whereas the detail for the XAI factors can be referred for pre-processing (Section 4.1 and Section 7.1), model selection (Section 4.2), model re-configuration (Section 7.2), interpretability (Section 1.2 and Section 4.3), explainability (Section 7), evaluation (Section 1.3 and Section 7.3), human-in-the-loop (Section 6.2), and XAI-RS (Section 10). It is recommended that the XAI should be evaluated every year to maintain the XAI system’s quality and validity.

**Figure 11:**
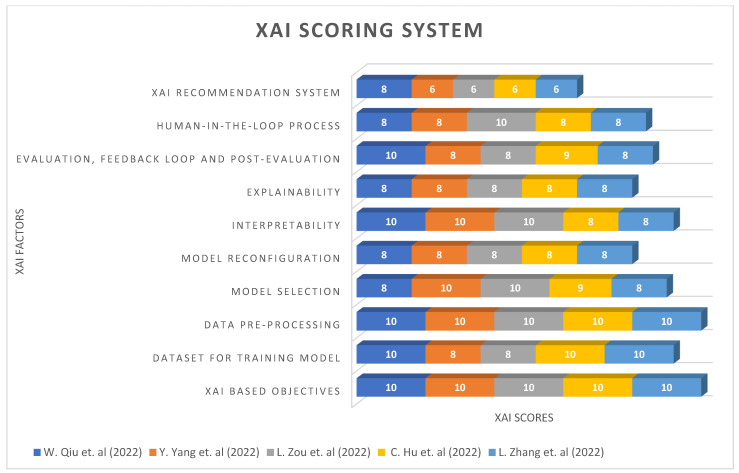
XAI Scoring System Evaluation.

The Table 10 Grades for the XAI scoring system in Figure 11 provides the evaluation for the recent XAI medical references [98]. It is helpful to analyze the applicability of XAI in the recent works and for the future works mapping.

## 12. Conclusions

The XAI survey presents a detailed approach for the XAI research development. Legal and ethical XAI aspects are presented, as well as some important areas within the medical field that need attention to improve it further and gain the user’s trust by providing transparency within the process. The contribution of XAI Recommendation System and XAI Scoring System will be suitable for overall development of XAI in the future. The future work will be focused on presenting the enhancements by XAI and further contributions as the recent progress is quite impressive.

## Figures and Tables

**Table 1:** General analysis for the explanation-based preprocessing.

Ref. #	Reference	Reference Paper/Mechansim	Data Preprocessing	Evaluation Methods/Algorithms	Outcome/Explanation Type
[11]	Selvaraju, R.R. et al. (2017)	GRAD-CAM/Global	GRAD-CAM	VGG, Structured CNN, Reinforcement Learning comparisons.	Textual explanations and AUROC/post-hoc
[12]	Tang, Z. et al. (2019)	Guided GRAD-CAM/Global	GRAD-CAM and feature occlusion analysis.	Segmentation on heatmaps and CNN scoring.	AUROC, PR curve, *t*-test and *p*-value/post-hoc
[13]	Zhao, G. et al. (2018)	Respond CAM/Global	GRAD-CAM, weighted feature maps and contours.	Sum to score property on 3D images by CNN.	Natural images captioning by prediction/post-hoc
[14]	Bahdanau et al. (2014)	Multi-Layer CAM/Global	Conditional probability	Encoder–decoder, neural machine translation and bidirectional RNN.	BLEU score, language translator and confusion matrix/post-hoc
[15]	Lapuschkin, S. et al. (2019)	LRP 1/Local (Layer-wise relevance propagation).	Relevance heatmaps.	Class predictions by classifier, Eigen-based clustering, LRP, spectral relevance analysis.	Detects source tag, elements and orientations. Atari breakout/ante-hoc
[16]	Samek, W. et al. (2016)	LRP 2/Local	Sensitivity	LRP, LRP connection to the Deep Taylor Decomposition (DTD).	Qualitative and quantitative sensitivity analysis. importance of context measured/post-hoc
[17]	Thomas, A. et al. (2019)	LRP DeepLight/ Local	Axial brain slices and brain relevance maps.	Bi-directional long short-term memory (LSTM) based DL models for fMRI.	Fine-grained temporo-spatial variability of brain activity, decoding accuracy and confusion matrix/post-hoc
[18]	Arras, L. et al. (2016)	LRP CNN/Local	Heatmap visualizations/PCA projections.	Vector-based document representations algorithm.	Classification performance and explanatory power index/ante-hoc
[19]	Hiley, L. et al. (2020)	LRP DNN/Local	Sobel filter and DTD selective relevance (temporal/spatial) maps	A selective relevance method for adapting the 2D explanation technique	Precision is the percentage overlap of pixels, std. and Avg. precision comparison/ante-hoc
[20]	Eberle, O. et al. (2020)	LRP BiLRP/Global	DTD to derive BiLRP propagation rules.	Systematically decompose similarity scores on pairs of input features (nonlinear)	Average cosine similarity to the ground truth, similarity matrix/ante-hoc

**Table 2:** Analysis for the BSI-affected patients treatment research.

Ref. #	Reference Paper	Dataset	Data Preprocessing/Mechanism	Evaluation Methods/Algorithms	Outcome/Explanation Type
[21]	Burnham, J.P. et al. (2018)	430 patients	Chi-squared/Fisher exact test, Student *t* test /Mann–Whitney U/Global	Multivariate Cox proportional hazards models	Kaplan–Meier curves and *p*-values/ante-hoc
[22]	Beganovic, M. et al. (2019)	428 patients	Chi-square/ Fisher exact test for categorical variables, and *t* test/ Wilcoxon rank for continuous variables./Global	Propensity scores (PS) using logistic regression with backward stepwise elimination and Cox proportional hazards regression model.	*p*-values./ante-hoc
[23]	Fiala, J. et al. (2019)	757 patients	Generalized estimating equations (GEE) and Poisson regression models/Global	Logistic regression models, Cox proportional hazards (PH) regression models	*p*-value before and after adjustment/ante-hoc
[24]	Fabre, V. et al. (2019)	249 patients	χ2 test and Wilcoxon rank sum test/Local	multivariable logistic regression for propensity scores	Weighted by the inverse of the propensity score and 2-sided *p*-value/ante-hoc
[25]	Harris, P.N.A. et al. (2018)	391 patients	Charlson Comorbidity Index (CCI) score, multi-variate imputation/Global	Miettinen–Nurminen method (MNM) or logistic regression.	A logistic regression model, using a 2-sided significance level
[26]	Delahanty, R.J. et al. (2018)	2,759,529 patients	5-fold cross validation/Local	XGboost in R.	Risk of Sepsis (RoS) score, Sensitivity, Specficity and AUROC/post-hoc
[27]	Kam, H.J. et al. (2017)	5789 patients	Data imputation and categorization./Local	Multilayer perceptron’s (MLPs), RNN and LSTM model.	Accuracy and AUROC/post-hoc
[28]	Taneja, I. et al. (2017)	444 patients	Heatmaps, Riemann sum, categories and batch normalization/Global	Logistic regression, support vector machines (SVM), random forests, adaboost, and naïve Bayes.	Sensitivity, Specificity, and AUROC/ante-hoc
[29]	Oonsivilai, M. et al. (2018)	243 patients	Z-score, the Lambda, mu, and sigma (LMS) method. 5-fold cross-validated and Kappa based on a grid search/Global	Decision trees, Random forests, Boosted decision trees using adaptive boosting, Linear support vector machines (SVM), Polynomial SVMs, Radial SVM and k-nearest neighbours (kNN)	Comparison of perfor-mance rankings, Calibration, Sensitiv-ity, Specificity, *p*-value and AUROC/ante-hoc
[30]	García-Gallo, J.E. et al. (2019)	5650 patients	Least Absolute Shrinkage and Selection Operator (LASSO)/Local	Stochastic Gradient Boosting (SGB)	Accuracy, *p*-values and AUROC/post-hoc

**Table 3:** Analysis for the AKI-affected patients treatment research.

Ref. #	Reference	Dataset	Criteria	Data Preprocessing/Mechanism	Evaluation Methods/Algorithms	Outcome/Explanation Type
[31]	Lee, H-C. et al. (2018)	1211	Acute kidney injury network (AKIN)	Imputation and hot-deck imputation/Global	Decision tree, random forest, gradient boosting machine, support vector machine, naïve Bayes, multilayer perceptron, and deep belief networks.	AUROC, accuracy, *p*-value, sensitivity and specificity/ante-hoc
[32]	Hsu, C.N. et al. (2020)	234,867	KDIGO	Least absolute shrinkage and selection operator (LASSO), 5-fold cross validation/Local	Extreme gradient boost (XGBoost) and DeLong statistical test.	AUROC, Sensitivity, and Specificity/ante-hoc
[33]	Qu, C. et al. (2020)	334	KDIGO	Kolmogorov–Smirnov test and Mann–Whitney U tests/Local	Logistic regression, support vector machine (SVM), random forest (RF), classification and regression tree (CART), and extreme gradient boosting (XGBoost).	Feature importance rank, *p*-value and AUROC/ante-hoc
[34]	He, L. et al. (2021)	174	KDIGO	Least absolute shrinkage and selection operator (LASSO) regression, Bootstrap resampling and Harrell’s C statistic/Local	Multivariate Cox regression model and Kaplan-Meier curves.	*p*-value, Accuracy, Sensitivity, Specificity, and AUROC/ante-hoc
[35]	Kim, K. et al. (2021)	482,467	KDIGO	SHAP, partial dependence plots, individual conditional expectation, and accumulated local effects plots/Global	XGBoost model and RNN algorithm	*p*-value, AUROC/post-hoc
[36]	Penny-Dimri, J.C. et al. (2021)	108,441	Cardiac surgery-associated (CSA-AKI)	Five-fold cross-validation repeated 20 times and SHAP/Global	LR, KNN, GBM, and NN algorithm.	AUC, sensitivity, specificity, and risk stratification/post-hoc
[37]	He, Z.L. et al. (2021)	493	KDIGO	Wilcoxon’s rank-sum test, Chi-square test and Kaplan–Meier method/Local	LR, RF, SVM, classical decision tree, and conditional inference tree.	Accuracy and AUC/ante-hoc
[38]	Alfieri, F. et al. (2021)	35,573	AKIN	Mann–Whitney U test/Local	LR analysis, stacked and parallel layers of convolutional neural networks (CNNs)	AUC, sensitivity, specificity, LR+ and LR-/post-hoc
[39]	Kang, Y. et al. (2021)	1 million.	N.A.	conjunctive normal form (CNF) and Disjunctive normal form (DNF) rules/Global	CART, XGBoost, Neural Network, and Deep Rule Forest (DRF).	AUC, log odd ratio and rules based models/post-hoc
[40]	S. Le et al. (2021)	2347	KDIGO	Imputation and standardization/Global	XGBoost and CNN.	AUROC and PPV/post-hoc

**Table 4:** Analysis for the hospital mortality prediction research.

Ref. #	Reference	Dataset	Ventilator	Data Preprocessing/Mechanism	Evaluation Methods/Algorithms	Outcome/Explanation Type
[41]	Mamandipoor, B. et al. (2021)	Ventila dataset with 12,596	Yes	Mathews correlation coefficient (MCC)/Global	LR, RF, LSTM, and RNN.	AUROC, AP, PPV, and NPV/post-hoc
[42]	HU, C.A. et al. (2021)	336	Yes	Kolmogorov–Smirnov test, Student’s *t*-test, Fisher’s exact test, Mann–Whitney U test, and SHAP/Global	XGBoost, RF, and LR.	*p*-value, AUROC/ante-hoc
[43]	Rueckel, J. et al. (2021)	86,876	Restricted ventilation (atelectasis)	Fleischner criteria, Youden’s J Statistics, Nonpaired Student *t*-test/Global	Deep Neural Network.	Sensitivity, Specificity, NPV, PPV, accuracy, and AUROC/post-hoc
[44]	Greco, M. et al. (2021)	1503	Yes	10-fold cross validation, Kaplan–Meier curves, imputation and SVM-SMOTE/Global.	LR and Supervised machine learning models	AUC, Precision, Recall, F1 score/ante-hoc
[45]	Ye, J. et al. (2020)	9954	No	Sequential Organ Failure Assessment (SOFA) score, Simplified Acute Physiology Score II (SAP II), and Acute Physiology Score III (APS III)./Global	Majority voting, XGBoost, Gradient boosting, Knowledge- guided CNN to combine CUI features and word features.	AUC, PPV, TPR, and F1 score/ante-hoc
[46]	Kong, G. et al. (2020)	16,688	Yes	SOFA and SAPS II scores./Local	Least absolute shrinkage and selection operator (LASSO), RF, GBM, and LR.	AUROC, Brier score, sensitivity, specificity, and calibration plot/ante-hoc
[47]	Nie, X. et al. (2021)	760	No	Glasgow Coma Scale (GCS) score, and APACHE II/Global	Nearest neighbors, decision tree, neural net, AdaBoost, random forest, and gcForest.	Sensitivity, specificity, accuracy, and AUC/ante-hoc
[48]	Theis, J. et al. (2021)	2436	N.A.	SHAP, SOFA, Oxford Acute Severity of Illness Score (OASIS), APS-III, SAPS-II score, and decay replay mining/Global	LSTM encoder–decoder, Dense Neural Network.	AUROC, Mean AUROC and 10-FOLD CV AUROC/post-hoc
[49]	Jentzer, J.C. et al. (2021)	5680	Yes	The Charlson Comorbidity Index, individual comorbidities, and severity of illness scores, including the SOFA and APACHE-III and IV scores/Global	AI-ECG algorithm	AUC/post-hoc
[50]	Popadic, V. et al. (2021)	160	Yes	N.A./Local	Univariate and multivariate logistic regression models	*p*-values, ROC curves/ante-hoc

**Table 5:** XAI Human-In-The-Loop References.

XAI Category	Sub-Section	References
**Pre-Processing**	Dataset Consistency	[15,18,21,22,23,25,26]
	Imputation	[4,22,25,26,28,29]
	Data Distribution	[15,18,21,22,25,26,28,29]
	Image Registration	[4,5,17,19]
	Feature Scoring	[11,12,13,17,18,25,28]
	Feature Priority	[9,11,16,23,24,25,28]
	Equal Feature Scoring	[11,15,19,20,29]
	Threshold (Feature Selection)	[12,17,20,23,24,25,26]
	Manual Feature Selection	[12,24,25,26,28]
	Binary/Multi-Class Feature	[4,23,25,29]
**Methodology**	Feature Validation	[14,18,25,26,28]
	Novel Approach	[4,11,16,20]
	Method Inefficiency	[22,23,25,26,28]
	Feature Analysis	[11,18,25,26,27,28,29]
	Severity Level Analysis	[9,23,25,29]
	Feature Effectiveness	[18,20,23,25,28]
	Feature Averaging	[4,28,29]
	Feature Improvements	[16,17,24,25,29]
**Evaluation**	Model Metrics	[4,17,26,27,28,29]
	Classification	[11,12,14,17,27]
	Graphs	[12,16,18,25,27,28,29]

**Table 6:** Publicly available dataset for medical experiments.

Dataset Source	Medical Domain	Category	Size
RSNA(Radiological Society of North America) and NIH [72]	Pneumonia	NIH chest X-ray dataset with initial annotation.	26,601 CXR Images
Kermany [73]	Pneumonia	Chest X-rays	5856 CXR Images
Chest radiographs (SCR) dataset X-ray images [74]	Pneumonia	Chest radiographs	247 frontal viewed posterior-anterior (PA)
Central Line-Associated Bloodstream infections (CLABSI) in California Hospitals [75]	Blood Stream Infections (BSI)	The CLABSI (text/csv) dataset contains reported infections, baseline data predictions, days count for central line, standard infection ratio (SIR), associated confidence interval of 95%, and grading with respect to national baseline.	Details from 461 hospitals.
MIMIC Clinical Database [76,77]	Epidemics (HER Data)	The MIMIC dataset consists of ICU data with high patient’s count including vital signs, laboratory test, and medication courses.	The MIMIC-III database has 26 relational tables containing patient’s data (SUBJECT_ID), hospital admissions (HADM_ID), and ICU admissions(ICUSTAY_ID).
ICES Data Repository [78]	EHR Data	EHR Data recorded from the health services of Ontario.	13 million people.
Veterans Health Administration [79,80]	EHR Data	EHR data from US Veteran’s Affairs (VA) dataset	1293 health care facilities with 171 medical center and 1112 outpatient sites.

**Table 7:** XAI post-treatment recommendation chart.

1. Diet	2. Medicine/Treatment	3. Exercise	4. Regular Checkup	5. Side Effects
a. Fruits b. Vegetables c. Seafood d. Meat e. Grains f. Soup g. Milk Products	a. Morning dose 1 b. Afternoon dose 2 c. Evening dose 3 d. Lotions/Drops e. Physiotherapy f. Injections g. Dialysis	a. Walking/Running b. Yoga c. Cycling d. Swimming e. Sports	a. Daily b. Alternate day c. Weekly d. Bio-sensors/Remote health monitoring e. Monthly f. Quarterly/Year	a. Vomiting b. Dizziness c. Headache c. Loss of Appetite d. Skin rashes e. Palpitations

**Table 8:** XAI Personal Post-Treatment Recommendation Chart for AKI Patient.

1. Diet	2. Medicine/Treatment	3. Exercise	4. Regular Checkup	5. Side Effects
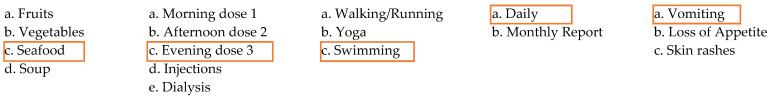				

**Table 9:** Checklist for XAI scoring system.

Serial No.	XAI Scoring Factor	Description	Checklist (10 pts Each)
1	XAI based Objectives	To serve the evaluation purpose for effective problem solving following laws and ethics.	
2	Dataset for Training Model	Whether the dataset has global and local scope?	
3	Data Pre-processing	Manage the data consistency and imbalance issue.	
4	Model Selection	To perform feature analysis and select an appropriate model with a novel approach.	
5	Model Reconfiguration	The model’s hyper-parameter tuning for better prediction by handling bias and variance.	
6	Interpretability	How much does the model support intrinsic and post-hoc interpretability?	
7	Explainability	Transparency in every step and decision of the model should be given by the algorithm.	
8	Evaluation, Feedback loop and Post-evaluation	The outcome should provide meaningful results. Graphs, prediction, and classification should be cross-verifiable. The feedback loop consisting of interacting with domain experts is helpful for post-evaluation.	
9	Human-in-the-Loop Process	Continuously involve the domain expert for improving multi-modal data and feature management.	
10	XAI Recommendation System	To maintain the discharged patient’s health conditions.	

**Table 10:** Grades for the XAI Scoring System.

Reference	XAI Scores	Grades
W. Qiu et al. 2022 [99]	90	Class I
Y. Yang et al. 2022 [100]	86	Class II
L. Zou et al. 2022 [101]	88	Class II
C. Hu et al. 2022 [102]	86	Class II
L. Zhang et al. 2022 [103]	84	Class II

**Figure A1:**
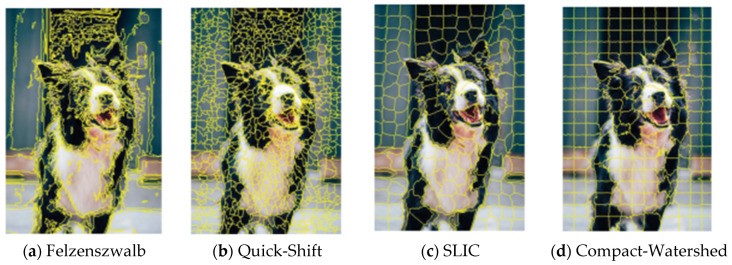
Comparison of super-pixel approaches.

**Figure A2:**
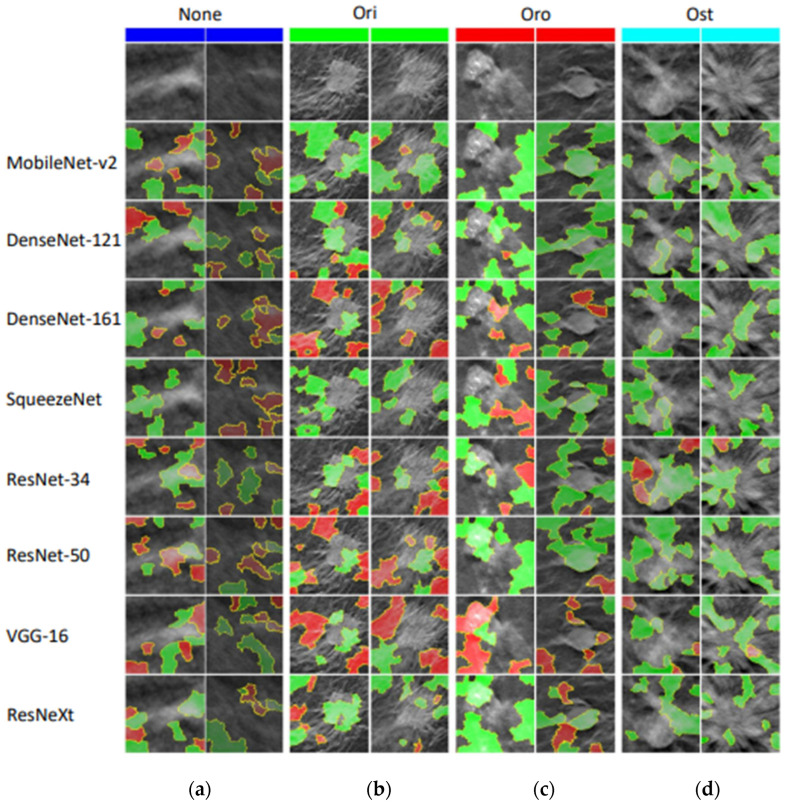
LIME Region of Interest (ROI) images for (**a**) No lesions (None); (**b**) Irregular opacity (Ori); (**c**) Regular opacity (Oro); and (**d**) Stellar opacity (Ost).

**Figure A3:**
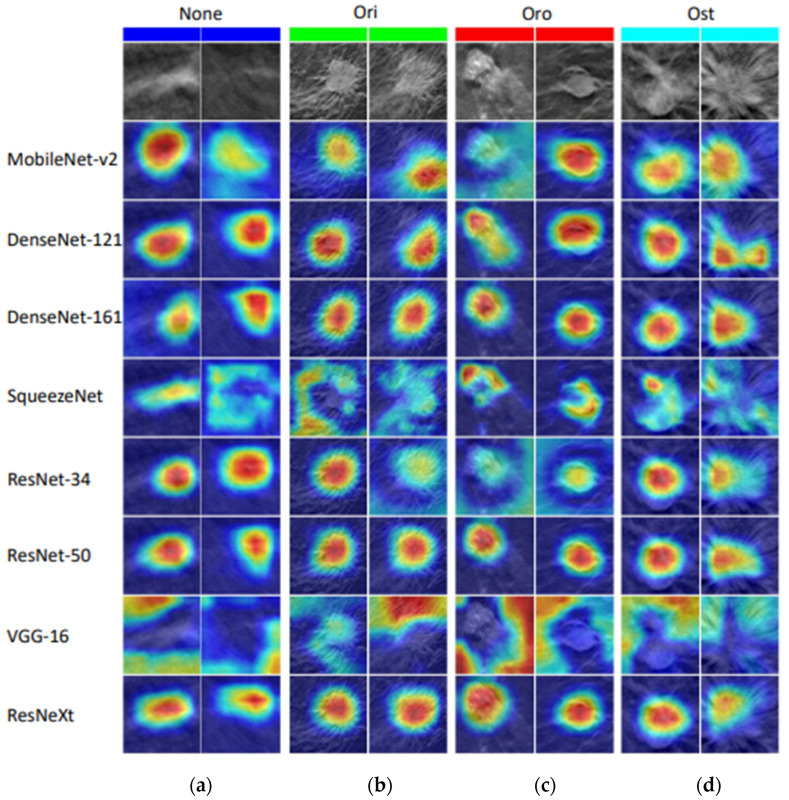
GRAD-CAM Region of Interest (ROI) images for (**a**) No lesions (None); (**b**) Irregular opacity (Ori); (**c**) Regular opacity (Oro); and (**d**) Stellar opacity (Ost).

## Data Availability

Not applicable.

## Appendix A

### Appendix A.1. LIME-Based Super-Pixel Generation

Pixels present the image in grid format and as a part of the original picture. Pixels are also known as artifacts that are meant to create digital images. In contrast, the originating source and semantic meaning of a specific super-pixel can be estimated [104,105]. A super-pixel is usually a pixel group or combination based on a set of common properties including pixel color value. The benefits of using super-pixel are given as: (a) Less complexity: The grouping of pixels based on distinct properties reduces the complexity and requires less computations. (b) Significant entities: A group of super-pixels having texture properties achieves an expressiveness through embedding. (c) Marginal information loss: In case of over-segmentation, crucial areas are highlighted with a minor deficit of less valuable data. Figure A1 shows the four types of super-pixel classification algorithms are explained as given below:

(i) Felzenszwalb and Huttenloch (FSZ) algorithm: The FSZ is a well-known algorithm for graph based approach and utilized for image edge operation with a O(M logM) complexity. The FSZ algorithm takes a weighted gradient with the same properties between two adjacent pixels. Successively, a future super-pixel seed is administered per pixel for obtaining the shortest gradient difference and largest for adjacent segments.

(ii) Quick-Shift (QS): QS is the default algorithm used by LIME. The QS algorithm generates super-pixels by mode seeking segmentation scheme. It then moves every point towards higher density leading to increased density.

(iii) Simple Linear Iterative Clustering (SLIC): The SLIC belongs to a cluster based super-pixel algorithm. It operates on the basis of *k*-means algorithm with a search space proportional size (*S* × *S*) for reducing distance calculations significantly.

$$D=\sqrt{d_{c}^{2}+{\left(\frac{d_{s}}{S}\right)}^{2}m^{2}}$$

The spatial $d_{s}$ and color $d_{c}$ proximity is combined by weighted distance measure having complexity of O(N). The size and compactness of super-pixel is given by m as given in the above equation. The algorithm initiates k cluster process as centers, which is grid scanned with S pixel’s distance with $S=\sqrt{\frac{N}{K}}$ approximately. To avoid super-pixel placement on the edge, the centers are shifted towards the smallest gradient. Later, each pixel is allotted to proximate clusters whose search area is overlaid on a super-pixel. Successively, the new cluster center is taken as pixels’ average vector for every center’s update. Finally, the residual error E is minimized until the threshold value and independent pixels are added to proximate super-pixel.

(iv) Compact-Watershed (CW): The CW algorithm is the optimized version of watershed super-pixel algorithm. The gradient image is used as input, where the altitude is the gray tone of each pixel that can be depicted as a topographical surface. The watershed with catchment basins are resulted from the continuous surface flooding, which may lead to over-segmentation and can be avoided by markers. The algorithm includes: (i) Flooding avoidable markers for each label, (ii) Marked areas neighboring pixels are collected in priority queue and graded by gradient magnitude equivalent to its priority level. (iii) Pixels with highest priority are pulled out and labeled according to their neighboring pixel. Later, labeled pixels are added in this queue. (iv) Step 3 is repeated until the priority queue is null.

The CW in terms of size and extensions has more compact super-pixels than watershed algorithms. To obtain this, the use of Euclidean distance for the difference with a pixel from super-pixel seed point by weighted distance measure and the gray value comparison within pixel and gray pixel’s value is performed.

### Appendix A.2. Perceptive Interpretability Methods

The XAI based perceptive interpretable methods [106,107] are LIME and GRAD-CAM, which are the CNN architecture-based decision explanation methods. The input given is a trained model for this interpretable process known as post-hoc analysis. The details of the XAI perceptive models for the shapes detection of cancerous masses in the breast imaging by computer-aided diagnosis (CAD) are as given below:

(a) Local Interpretable Model-Agnostic Explanations (LIME): The LIME interpretation is provided by highlighting the top contributing of class S, which is evaluated on an image classification by observing a ground truth prospect. Figure A2 shows the S perturbations executed by LIME, which are similar to GRAD-CAM, whereas Figure A3 GRAD-CAM is using the same image for comparison with LIME in Figure A2. The ground truth class is used for the CNN based prediction is the same class used for LIME perturbations. The green color indicates positive correlation of regions with the CNN decision, and red color indicates negative correlation. Figure A2 shows the saliency scheme is used for the presentation of saliency zones, where higher intensity red color focuses more for classification and lower intensity blue color has less focus. Several classes are distinguished by header bars with distinct colors.

(b) Class Activation Mapping (CAM): The Figure A3 shows the GRAD-CAM methods based pictorial presentation of eight fine-tuned networks for visual explanation. The figure includes for every class, two sample images are presented and every network’s respective saliency maps for the images. The links within lesion area and network performance are highlighted for which accurate prediction of images is given by the network. The ground truth is considered for generating saliency maps of the approximate features. Subsequently, the lesion areas which are incorrectly identified by CN architectures are also affected during classification tasks. In case of SqueezeNet, the AUC is not significant and AUC trade-offs with the number of parameters obtained from VGG-16 are incapable of highlighting the lesion area, whereas, the lesion of the images is accurately highlighted by the ResNet-50, DenseNet-121, and DenseNet-161. Therefore, the GRAD-CAM is distinct from LIME as it emphasizes the color intensity closer to the center of the lesion area. Figure A3 presents the super-pixels with red color for negative contribution and green color otherwise. Similarly, several classes are distinguished by header bars with distinct colors. Samples belonging to the same class are represented by similar images header color.

Thus, it can be noticed that graphical reasoning has good understanding in the CAM-based interpretations, whereas the evaluation of LIME and GRAD-CAM activation maps are completely different which can be noticed from Figure A2 and Figure A3. Ultimately, it is recommended to consider both interpretations and models evaluation to gain a broader view of this process.
